# A Comprehensive Assessment on the Pivotal Role of Hydrogels in Scaffold-Based Bioprinting

**DOI:** 10.3390/gels8040239

**Published:** 2022-04-13

**Authors:** Matangi Parimala Chelvi Ratnamani, Xinping Zhang, Hongjun Wang

**Affiliations:** 1 Department of Biomedical Engineering, Stevens Institute of Technology, Hoboken, NJ 07030, USA; mpr@stevens.edu; 2 Department of Orthopaedics, Center for Musculoskeletal Research, University of Rochester Medical Center, Rochester, NY 14642, USA; xinping_zhang@urmc.rochester.edu; 3 Department of Chemistry and Chemical Biology, Stevens Institute of Technology, Hoboken, NJ 07030, USA

**Keywords:** 3D bioprinting, living cells, hydrogel matrix, layer-by-layer assemblage, hydrogel characterization, bioprinting limitation, strategies

## Abstract

The past a few decades have seen exponential growth in the field of regenerative medicine. What began as extirpative (complete tissue or organ removal), with little regard to the effects of tissue loss and/or disfigurement, has evolved towards fabricating engineered tissues using personalized living cells (e.g., stem cells), and customizing a matrix or structural organization to support and guide tissue development. Biofabrication, largely accomplished through three-dimensional (3D) printing technology, provides precise, controlled, and layered assemblies of cells and biomaterials, emulating the heterogenous microenvironment of the in vivo tissue architecture. This review provides a concise framework for the bio-manufacturing process and addresses the contributions of hydrogels to biological modeling. The versatility of hydrogels in bioprinting is detailed along with an extensive elaboration of their physical, mechanical, and biological properties, as well as their assets and limitations in bioprinting. The scope of various hydrogels in tissue formation has been discussed through the case studies of biofabricated 3D constructs in order to provide the readers with a glimpse into the barrier-breaking accomplishments of biomedical sciences. In the end, the restraints of bioprinting itself are discussed, accompanied with the identification of available engineering strategies to overcome them.

## 1. Introduction

Three-dimensional (3D) bioprinting, a subcategory of the additive manufacturing (AM) technology, is a novel technique brought forth by the convergence of biology and material sciences within the regime of 3D printing. Here, the re-creation of living tissues is facilitated through the use of hydrogels as the ink, together with cells and other relevant bioactive molecules, from which complex tissue structures are printed [1]. With the growing global shortage of organs and limited organ-donor availability, the principles of tissue engineering are integrally transformed with technological advancements such as 3D printing to create human-made biological substitutes as permanent solutions for tissue loss or damage.

Conventionally, tissue engineering strategies utilizing scaffolds as matrices for tissue formation are limited due to (i) their inefficacy for cell seeding and penetration, (ii) the lack of orchestrated cell positioning, and (iii) the absence of vascularization [2]. In this regard, bioprinting, which aims to partially if not fully address these limitations, enables (i) a better recapitulation of the cellular microenvironment, vasculature, and biological organization via a customized bioink formula and computer-aided deposition, (ii) spatially controlled cell distribution through heterogenous cell printing to capture the in vivo compositional complexity, and (iii) guided cell differentiation and tissue development via the relevant intracellular interactions [3]. So far, bioprinting has been explored to create various tissue models (e.g., cardiac tissue patches [4], osteochondyle [5], solid tumors [6], etc.) for in vitro utilities and in vivo implantations. Several successful 3D in vitro models established through bioprinting include the development of miniature kidneys used for screening drug toxicity [7], and hepatic lobule-like spheroid models displayed prolonged viability with a uniform size in the micrometer range [8]. Three-dimensional heart models were able to duplicate the electromechanical functionality and calcium wave propagation, along with peristaltic valve opening and closing [4], and a 3D-printed ear auricle showed excellent reproducibility of audio signals [9]. As a result of the computer-aided deposition, it becomes highly feasible to fabricate the tissue constructs with a great degree of versatility, spatial organization, and structural precision. Meanwhile, the possibility of controlling porosity and channel sizes in the printed constructs allows for mass exchange and vascularization, which are essential for tissue regeneration.

### 1.1. Bioprinting Strategies

Bioprinting typically employs three primary strategies, namely, biomimicry, autonomous self-assembly, and microtissue building blocks. In *biomimicry*, the functional components of cells are reproduced following the cellular interactions with internal and external influencers such as signaling molecules, soluble and insoluble factors, temperature, pressure, etc. [10]. Biomaterial scaffolds are used as the supporting structures [10]. Each key step of the tissue development can theoretically be duplicated by controlling the spatial positioning of heterogenous cells. However, the replication of all necessary biological cues for cell growth and development can be laborious [10]. *Autonomous self-assembly* uses the embryonic organ-development process as a template to form the biological tissues [10]. Cellular spheroids, with the correct embryonic elements in place, undergo self-assembly and cell fusion to recapitulate the in vivo tissue development process [10]. However, the self-assembly is limited by the difficulties associated with structural changes during or after the commencement of organization [10]. Cells, the basic building blocks of an organism, make up the tissues that conform into specialized organs. *Microtissue or mini-tissue building blocks* rely on this hierarchical assemblage for engineering tissue constructs [3]. The smallest structural and functional units of a tissue are developed, which are later conjugated to form the target tissues [10]. The difficulty with large-volume tissue-engineered constructs is the lack of a functional vascular network, limiting oxygen and nutrient supplies from surrounding culture medium; relying on the solute diffusion from the medium can be only effective up to a distance of 200 μm [11,12]. Although the mini-tissue building blocks help circumvent this shortcoming in in vitro 3D constructs, the accurate creation of the structural building blocks at the micro-scale level remains a challenge [3].

### 1.2. Bioinks

Biofabrication makes use of aggregate emulsions, called bioinks, containing living cells suitable for bioprinting or bio-assembly. These emulsions may also accommodate biologically active components such as drugs, growth factors, and/or biomaterials [13]. Bioinks can be both scaffold-based and scaffold-free, depending on the presence or absence of exogenous support biomaterials within the bioink formula. Scaffold-free bioinks are composed entirely of cells and their secreted ECMs, whereas scaffold-based bioinks incorporate biomaterials as a supporting matrix to aid in cellular growth, proliferation, and differentiation. Scaffold-based bioinks will be prioritized in this review, with particular attention to the roles of hydrogels in cell printing.

#### 3D Printing Mechanism

**Figure 1:**
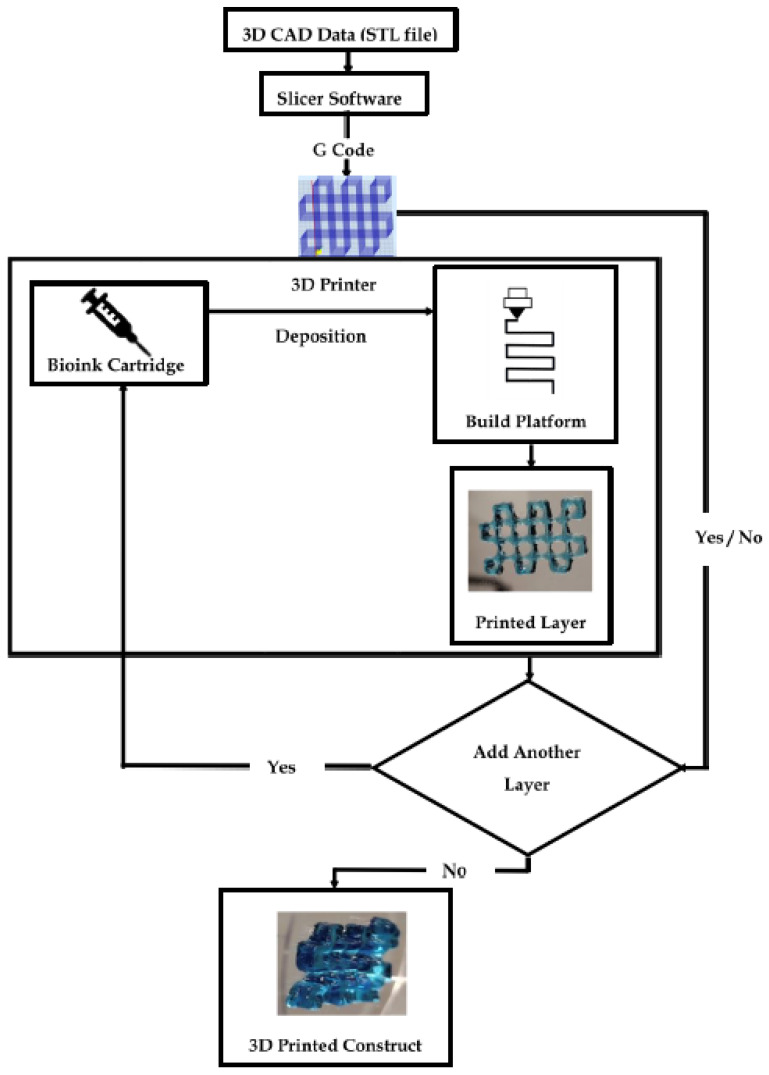
Illustration of a 3D bioprinting workflow.

In 3D printing, computer-aided design (CAD) data are used to direct hardware (3D printers) to deposit successive layers of a base material with precise geometries. Three-dimensional CAD data, generated from the imaging data of biological specimens such as X-rays, CT scans, or solid-modeling computer programs such as Solidworks, are generally saved in an STL (standard tessellation language or stereolithography) format and then fed to a slicer software. The slicer mathematically divides the 3D model into individual horizontal 2D layers or slices recognizable by the 3D printer. These slices will be printed one at a time, in a layer-by-layer fashion by the 3D printer. The slicer data, along with printer-specific commands such as printing temperature, speed and direction of movement of the printer nozzle, thickness of each printed layer, and printing path are compiled into an instructional geometric code (G-code) by the slicer, which is recognized by the 3D printer. Figure 1 captures the key steps of a typical bioprinting process.

The polymer cartridge is a reservoir of the printing material of choice (i.e., bioink). Based on the instruction specified by the G code, the bioink is deposited onto the working plate or build platform in a layer-by-layer fashion. The deposition may be assisted by laser beams, ultraviolet (UV) or visible light, or thermal, piezoelectric, or pneumatic forces [14]. The fusion of bioink layers is aided by the hydrogel gelation mechanism. Certain polymers used in biofabrication can undergo a sol–gel transition in the presence of light (e.g., polyethylene glycol) or variations in temperature (e.g., gelatin), pH (e.g., collagen), or ionic interactions (e.g., alginate) that help in the fusion of the subsequent layers until the 3D construct is complete [15,16,17].

### 1.3. Representative Bioprinting Techniques

Bio-fabrication is carried out via 3D printers that allow for the control of parameters such as the size, shape, cell positioning, and porosity of the printed construct through the manipulation of printer-specific commands that are set pre-printing. The properties of a bioink before, during, and after gelation dictate the choice of printing method. Characteristic profiles of a few biomaterials are detailed in Section 3.1.1, Section 3.1.2 and Section 3.1.3. Table 1 provides a concise outlook of the current bioprinting techniques employed. A detailed analysis can be found in [3,13,14,18,19,20,21].

**Figure 2:**
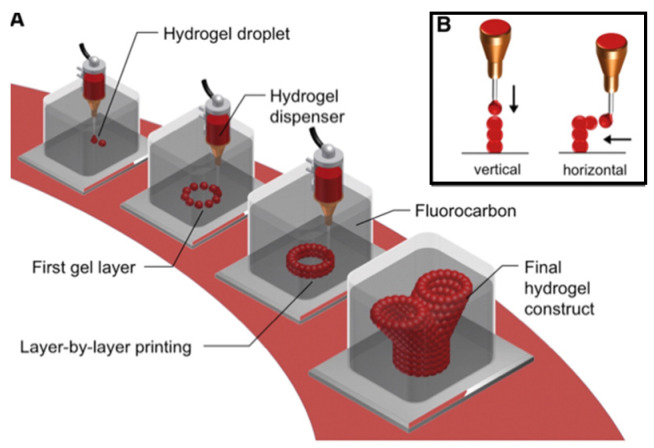
Schematic illustration of submerged bioprinting. (**A**) Droplet deposition of hydrogels within the submerged support matrix. (**B**) The buoyancy offered by the support matrix enables both vertical and horizontal deposition without collapse. Reproduced from Andreas Blaeser et al. [29].

The shape fidelity of soft and easily collapsible hydrogels during and after printing can be improved by depositing them into a thermo-reversible hydrogel supporting bath [26]. Freeform reversible embedding of suspended hydrogels (FRESH), or submerged printing (Figure 2), makes use of supporting baths that offer little mechanical resistance to the movement of the printing nozzle, behaving like a rigid body at low shear stresses but flowing like a viscous fluid at a higher shear stress [27,28]. Thermosensitive hydrogels such as agarose and gelatin can be used as the supporting matrix along with high-density molecules such as perfluorocarbons (PFCs) [16,27,29,30].

## 2. Hydrogels in Bioprinting

Hydrogels are 3D systems comprising physical or chemically bonded polymers that can be mixed with cells and biologically active molecules in an aqueous solution to form cell-laden crosslinked matrices [31]. They are predominantly hydrophilic and capable of absorbing an amount of water equal to 10 to 1000-times their dry weight [32]. A high water content imposes little diffusion resistance to solutes, oxygen, and nutrients, enabling hydrogels to mimic the natural soft tissue more than any other class of polymeric materials. This property, together with the good biocompatibility, processability, adaptability, the ability to provide functional cues to guide cellular assembly, and manipulatable tissue-matching degradation rates makes hydrogels attractive candidates for bioprinting applications [32,33].

### 2.1. Hydrogel Crosslinking Techniques

Hydrogel networks are formed by crosslinking the polymer chains in an aqueous medium. In contrast to linear polymers, crosslinked polymers become insoluble while retaining their swelling properties of their respective monomers [34]. The crosslinking of hydrogels by physical, chemical, or enzymatic mechanisms effectively immobilizes them, and aid in the release of bioactive agents such as drugs and growth factors trapped within their matrix. Temperature, pH, and inherent polymer electrostatic interactions are the key contributors of physical crosslinking [35]. Both chemical and enzymatic crosslinking make use of external agents known as crosslinker molecules (e.g., genipin, glutaraldehyde), photosensitive agents (e.g., Irgacure, lithium phenyl-2,4,6-trimethylbenzoylphosphinate (LAP)), or enzymes (e.g., tyrosinase, transglutaminase) [15]. A comparative summary on the hydrogel crosslinking techniques can be found in Table 2.

In physical crosslinking, polymer crosslinks occur by physical entanglements that are reversible and do not require chemical modifications to the polymer chains [36]. Hydrogels can transition from solution to gel state under the influence of increases (e.g., elastin, collagen, Matrigel, agarose) or decreases (e.g., gelatin) in temperature [35] and pH [16], or through inherent electrostatic attractions between charged polymer chain, e.g., alginate (anion) crosslinked with calcium ions or chitosan (cation) [32,37].

Chemical crosslinking procedures using exogenous crosslinking agents such as free radicals, photoinitiators (e.g., eosin Y, rose bengal (activated by green light), vitamin riboflavin (initiated by blue light), and Irgacure (activated by UV light)), and chemicals such as genipin and glutaraldehyde are used to induce the covalent bonding between polymeric chains (e.g., hyaluronic acid, gelatin methacrylate, silk) [15]. In addition, monomers with complementary chemical groups or with activated double bonds can react together to produce a multifunctional polymer (e.g., Michael addition) [15], or chemical groups within the monomers can be functionalized with azide or alkyne functional groups and “clicked” in the presence of a copper ion catalyst (click chemistry) [15,38]. Unlike physical crosslinking, the degree of chemical crosslinking and, thereby, the density of the polymer mesh can be controlled by the amount of crosslinker added or the number of reactive functional groups on the polymer chain. Hence, depending on the degree of cross-inking and the size of the bioactive molecules, the release of such bioactive molecules from hydrogel can be tailored [38,39].

Enzyme catalysis typically occurs at a neutral pH and a moderate temperature in aqueous environments, similar to the in vivo microenvironmental conditions. Transglutaminase can crosslink fibrin, elastin, and polyethylene glycol (PEG) by forming covalent bonds between free amine groups and γ-carboxamide groups in polymers [33]. In the presence of oxygen, tyrosinase acts on phenol-containing side chains to form quinones which can bond with materials having hydroxyl or amino groups. Gelatin and chitosan can be crosslinked using tyrosinase. Lysyl oxidase crosslinks collagen by oxidizing primary amines of lysine to aldehydes [33].

### 2.2. Nutrient Transport in Hydrogels

In addition to acting as cell-support structures, hydrogels must also facilitate nutrient and oxygen supply to the encapsulated cells. In vitro, nutrient transport occurs through convection (fast transport, due to bulk fluid movement) or diffusion (slow molecular movement in the direction of the concentration gradient), or a combination of both [11].

Tissue-engineering cultures are often static, with no active perfusion. Hence, the contribution of convection is ignored. The transport of nutrients can, thus, be described using Fick’s law of diffusion [11]:

(1) $$J=-D\;\frac{\mathrm{dc}}{\mathrm{dx}}$$

The negative sign indicates that the diffusion occurs in the direction opposite to the concentration gradient. Particulate movement due to diffusion is from high to low concentration, but the concentration gradient that causes them to migrate is negative; hence, to make J positive, the negative sign is used to compensate for the negative gradient.

J—flux.
D—diffusion coefficient of solute (nutrient and/or oxygen) in the medium.
c—concentration of the solute (nutrient and/or oxygen) in the medium.
x—distance.

In the cases where convective flow is not dominant, strategies to enhance mass nutrient transport within tissue constructs can be achieved by improving the diffusivity coefficient (D), through the provision of a hydrogel matrix with open channels for easy nutrient and oxygen diffusion; or by reducing the diffusion distance, using oxygen carriers or oxygen generators [11,40,41,42,43,44,45,46,47,48].

### 2.3. Hydrogel Swelling Kinetics

Solvent molecules interact with hydrogels when in contact, and penetrate through the polymer network, causing the hydrogels to swell. The swelling is not a continuous process [38]. Against the favorable osmotic force that enables solvent diffusion, there is an opposing elasticity force that balances the stretching of the polymer network and prevents its deformation [38]. At equilibrium, there is a balance between the osmatic and elastic forces, and no additional swelling occurs. Thus, the swelling data can be used to determine the crosslinking degree, the mechanical and viscoelastic properties, and the degradation rates of hydrogels [38].

The swelling rate of hydrogels is dependent on the porosity and the type of pore structure. Based on the pore morphology, hydrogels can be non-porous (pore size: 10–100 Å), micro-porous (pore size: 100 to 1000 Å), macro-porous (pore size: 0.1 to 1 μm), and super-porous (pore size: several-hundred-micrometer range) [38]. In the super-porous structures, the pore units within the hydrogel are connected to form open-channel systems that act as a capillary network, enabling rapid water uptake. The pore size classification, in turn, determines the rate of solute diffusion [38].

### 2.4. Biological Properties of Hydrogels

#### 2.4.1. Biocompatibility

Biocompatibility describes the tolerance between a technical (engineered) and a biological system. During biofabrication, cell viability is dependent on the printing technique employed, i.e., based on the printing process, certain hydrogel properties may affect the cells encapsulated within their matrix, e.g., the thermo-conductive nature of hydrogels used in thermal inkjet printing and the viscosity profile of extrusion-printed hydrogels may cause temperature variance and shear stress to the cells [16].

After printing, a cell–biomaterial interaction through adhesion decides the ability of cells to maintain their phenotype and proliferate [16]. The presence of innate cell-binding motifs of natural polymers provides an engaging microenvironment for cells to maintain their activities by mimicking the in vivo conditions [16].

Upon in vitro maturation, the immune response to hydrogels in vivo (immunogenicity) is dictated by the nature of the polymer making up the hydrogel [16]. Naturally derived biomaterials are susceptible to the acquired immunity, eliciting a target (antigen)-specific reaction, while synthetic biomaterials are subjected to the non-specific foreign body responses [16].

#### 2.4.2. Biodegradation

Hydrogels within the bioinks are temporary scaffolds that provide initial mechanical support for cell growth, up until the embedded cells begin to synthesize their own 3D supporting matrix, i.e., the ECM. In this regard, the rate of hydrogel degradation should, ideally, match with the rate of ECM synthesis.

If the rate of polymer degradation is slower than that of ECM formation, disruption to the normal tissue development and oxygen supplies are expected. Meanwhile, a slower degradation might also elicit certain immune responses. On the contrary, a too-fast degradation of hydrogel might unexpectedly deprive cells of their support anchorage, causing the loss of their shape, thereby impeding tissue development [16].

At the time of polymer degradation, the degradation mechanism and associated by-products should not cause any harm or damage to the regenerating tissue and/or surrounding tissues. Preferably, the degraded by-products from a hydrogel would be water-soluble and non-toxic for ready clearance. The degradation of polymers occurs primarily through enzymes (natural polymers), hydrolytic reactions (synthetic polymers), or ion exchange (charged polymers) [16]. Matrix-remodeling proteases, secreted by cells within the bioink formula, also contribute to the polymer degradation.

Degradation kinetics depend on the polymer type, its concentration, degree of crosslinking, cell type, and the cell density of the bioink [16]. To maximize cell development and proliferation, controlled hydrogel degradation can be achieved through the modulation of parameters such as (i) the cell–polymer ratio (to control the amount of matrix proteases secreted by cells); (ii) the degree of crosslinking (i.e., to increase the amount of crosslinkers and decelerate the degradation); (iii) the incorporation of inhibitor molecules (e.g., galardin for fibrin bioinks) within hydrogels to delay the enzyme-mediated degradation [16].

### 2.5. Rheological Properties

The viscosity and shear thinning (thixotropy) properties of hydrogels are important parameters to consider while bioprinting, since the bioink must be self-supportive post-printing whilst maintaining its fluidity during the printing process.

#### 2.5.1. Viscosity

Viscosity refers to a material’s resistance to flow in the presence of an applied force [23]. Depending on the printing techniques employed and the nature of the applied force, the viscosity requirements can vary. Inkjet printers manipulate thermal or acoustic pulses to deposit bioink droplets; hence, they require low-viscosity hydrogels. Extrusion printers apply pneumatic pressure to unload a continuous filament of bioink material that is of high viscosity, while laser-based printers, being nozzle-free, can employ hydrogels with a wide range of viscosities [3,19]. Hydrogels can be optimized for nozzle-based dispersion using Poiseuille’s equation [16]:

(2) $$\delta P=\frac{8\mu \mathrm{LQ}}{{\pi r}^{4}}$$

δP—difference between the applied pressure on the bioink and the ambient pressure.
L—length of the nozzle tip.
Q—flow rate or scan speed.
μ—viscosity of the bioink.
r—radius of the nozzle tip.

#### 2.5.2. Shear-Thinning

The protection of cellular components during bioprinting is necessary, as the fabrication process is usually accompanied by imposing pressure and shear forces, all of which can affect the cellular viability within the bioink. Hence, the hydrogel material must exhibit certain protections to the cells encapsulated within its matrix.

Shear-thinning is a polymer characteristic enabling the polymer to stretch and become more uniformly aligned in a direction parallel to the applied stress, thereby decreasing its viscosity for ease of flow across the printer nozzle. Thixotropy is a special case of shear-thinning, wherein the material behaves as a solid under low shear conditions and undergoes a quick transition to its liquid state once a critical shear strain is reached [43]. Due to this phenomenon, hydrogels may have a high viscosity within the bioink cartridge and post-deposition, while possessing a low-viscosity transition state for extrusion out of the printer nozzle without causing pressure to the cells within [43].

## 3. Hydrogel-Based Bioinks

**Figure 3:**
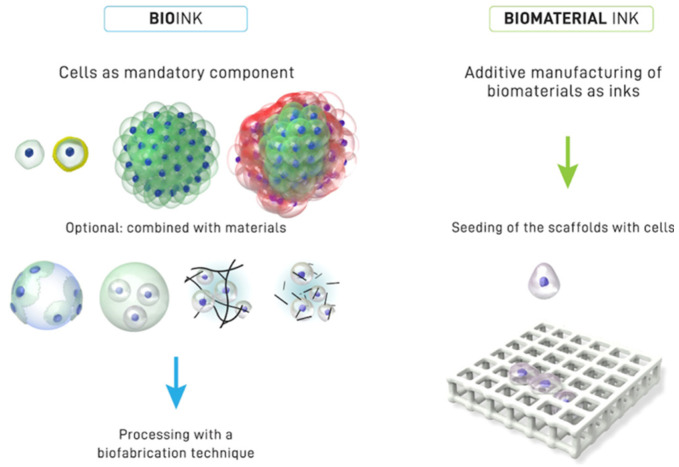
Distinction between bioink and biomaterial ink. Reproduced from Groll J. et al. [13].

The “bio” in bioinks typically refers to cells; hence, they are a mandatory component of bioinks. A formulation that contains biologically active molecules and biomaterials but not cells does not qualify as a bioink; instead, it would be considered a biomaterial ink [13] (Figure 3). This terminology applies to the case of 3D printing biomaterial scaffolds and then seeding them with cells. A biomaterial ink can be converted to a bioink through the inclusion of cells into the mixture pre-printing [13].

An ideal bioink must possess the desired physicochemical properties (e.g., mechanical, rheological, chemical, and biological characteristics) [43,44], leading to self-supporting tissue constructs with adequate mechanical strength and robustness.

The long-term viability of a bioprinted construct, especially post-transplantation, hinges on the choice of cell source [10]. The cells must be able to maintain their homeostasis and functionality and withstand physical challenges encountered during printing, such as heat, shear stress, and pressure, in addition to the chemical and biological stress factors such as toxins, enzymes, and pH. They should also be able to replicate the physiological microenvironment, self-renew, and respond to tissue damage or injury in vivo [10].

### 3.1. Classification of Scaffold-Based Bioinks

Hydrogel-based bioinks can be broadly classified based on their ultimate role in the printed construct and origin of the biomaterials. Based on the roles they play post-printing, hydrogel inks can be supportive, fugitive (sacrificial), structural, and functional. Their properties are detailed in [13]. Depending on the original source of the biomaterials incorporated within the bioink, the hydrogel-based bioinks can be classified as natural bioinks, synthetic bioinks, or a combination (hybrid inks) that possesses favorable properties of both natural and synthetic components [19]. Table 3 and Table 4 provide a concise summary on the natural, synthetic and hybrid bioinks, and their roles in biofabrication. These details are further elaborated in Section 3.1.1, Section 3.1.2 and Section 3.1.3.

#### 3.1.1. Natural Bioinks

##### Type 1 Collagen

Collagen is the main structural protein in the ECM of mammalian cells, with type I accounting for 90% of the total body collagen [16]. The collagen molecule arranges itself into a quaternary structure in a hierarchical fashion that extends from a basic collagen molecule (1.5 nm diameter) to a collagen fibril (30–300 nm diameter) [16]. Type I collagen is an amphoteric (i.e., acts as both an acid and a base due to the presence of anionic and cationic groups), rigid, triple-helical structure having polar and hydrophobic amino acid groups. The innate integrin-binding sites of collagen promote the attachment and proliferation of cells within the bioink, along with its tissue-matching physiochemical properties, superior biocompatibility, and low immunogenicity [16].

Collagen hydrogel can be prepared from the stock solutions derived from bovine skin or rat tendon that are chilled at 4 °C and dissolved in an acid solution to prevent precipitation [16]. For cellular encapsulation, the acidic solution of collagen needs to be neutralized at low temperature prior to cell incorporation. Incubating the chilled solution at 37 °C and 7.4 pH for 15 to 30 min gives the viscous, self-assembled collagen hydrogels [16]. Crosslinking collagen increases its tensile strength, viscoelastic properties, and printability. Crosslinking can be achieved using vitamins such as riboflavin through photopolymerization, thermal gelation, and chemical modification using glutaraldehyde, formaldehyde, or carbodiimide groups [45].

The mechanical properties and gelation times of collagen can be further optimized through the utilization of enzymes such as transglutaminase, lysyl oxidase, tyrosinase, and polyethylene glycosylation [16]. Un-crosslinked collagen can be used for inkjet printing due to its low viscosity, while the partially or fully crosslinked ones can be used for extrusion printing [16]. Collagen-based bioprinting finds its applications in soft to hard tissues such as skin [49], cartilage [50], bone (in conjunction with a scaffold support) [51], liver tissue (as a hybrid bioink with hyaluronic acid) [52], nerve regenerative models [53], corneas (with sodium alginate) [54], etc. As a representative example, a collagen-based heart structure [4] is detailed below.

Collagen Bioprinted 3D Heart Model:

Collagen solutions containing cardiomyocytes were used to create various components of the heart, from capillaries to a full organ [4] The FRESH printing technique was used to improve the printing fidelity.

**Figure 4:**
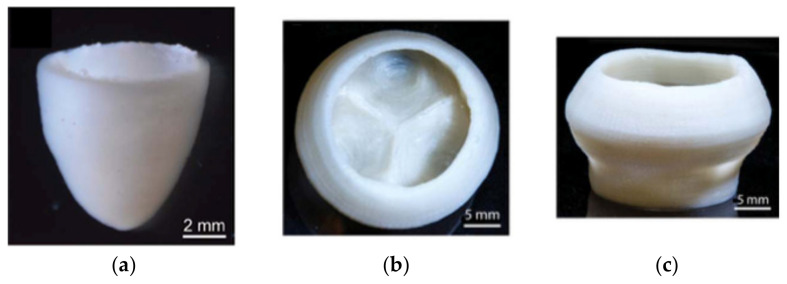
(**a**) A 3D-printed ventricle. Scale bar = 2 mm. Top (**b**) and side (**c**) views of 3D-printed tri-leaflet heart valve. Scale bar = 5mm. Reprinted with permission from [4]. Copyright (2019), *Science*.

The left ventricle of a heart model with a cell-free collagen outer wall and a cardiomyocyte-laden core was printed (Figure 4a). A 96% cell viability was achieved post-printing, and the printed construct was further cultured for 28 days. On day 4, ventricular contraction was visible, and after 7 days, the ventricle became synchronous with a dense layer of interconnected and striated cardiomyocytes. The printed ventricle also had good electromechanical functionality with a baseline spontaneous beat rate of ~0.5 Hz.

**Figure 5:**
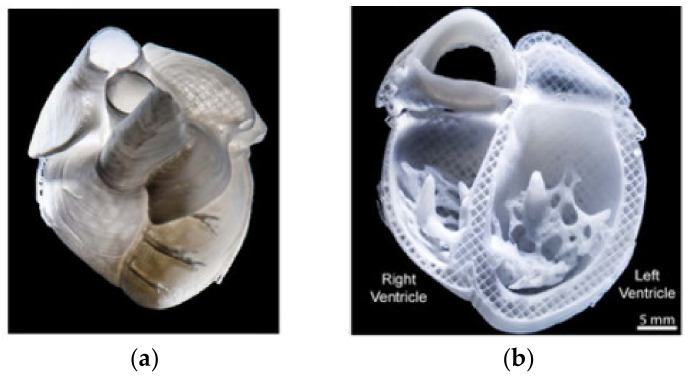
Bioprinted heart structure. (**a**) FRESH-bioprinted collagen heart. (**b**) Cross-sectional view of bioprinted heart showing right and left ventricles. Scale bar = 5 mm. Reprinted with permission from [4]. Copyright (2019), *Science*.

A tri-leaflet heart valve of 28 mm diameter (Figure 4b,c) and a neonatal human heart with a micro-scale internal structure (Figure 5) were also similarly printed from collagen. When subjected to a flow, opening and closing of the valve leaflets were observed with a <15% regurgitation and a maximum opening area of 19.5%.

##### Fibrin

Fibrin has a randomly arranged fibrillar network, allowing for the gradual release of soluble factors to aid in tissue regeneration [46]. Fibrin hydrogels are formed via the enzymatic catalyzation of the glycoprotein fibrinogen by thrombin in the presence of calcium ions (Ca^2+^). In vivo, thrombin activates factor XIII to reinforce the crosslinked structure. In vitro, genipin is often used to form the robust gels [46,47].

The fibrin structure has multiple cell adhesion peptide domains, such as arginine–glycine–aspartic acid (Arg–Gly–Asp or RGD), and its ability to associate with heparin, fibronectin, and integrin favor cell binding [47]. Fibrin is insoluble, biocompatible, biodegradable, non-immunogenic, and can induce cell proliferation and ECM formation [47]. Fibrin biodegradation in vivo takes place through plasma-mediated fibrinolysis by the serum protease plasmin, allowing it to be replaced by cell-secreted ECM and subsequently re-integrate within the cellular microenvironment of the newly formed tissue [46]. The rate of in vitro fibrinolysis can be tuned through the addition of protease inhibitors such as ε-aminocaproic acid, tranexamic acid, aprotinin, and galardin to mechanically reinforced fibrin, and can help preserve the intactness of the matrix for several weeks [16,47].

In vivo, fibrin clot degradation begins within a few hours of injury [16]. Similarly, fibrin hydrogels’ dissolution depends on the time (usually one week) taken for the cells to produce degradation enzymes. Hence, without an effective enhancement mechanism, fibrin gels lack structural and mechanical stability for tissue-engineering applications [16,47].

Increasing the calcium-to-thrombin ratio favors lateral aggregation over longitudinal polymerization, leading to thicker, shorter fibers. Contrastingly, a reduction in the calcium ratio gives rise to thinner, longer fibers [46]. Increasing the fibrinogen concentration causes a reduction in the diameter of individual fibers due to tighter fiber packing, along with a reduced pore size and nutrient permeability, i.e., a higher fibrinogen concentration produces thinner gels [48]. However, increasing the fibrinogen concentration adversely affects the fibrin stiffness and pore size. Hence, fibrinogen concentration is the determinant for the final fibrin stiffness [46].

The fibrin gels have a large stretchability and high elastic deformation capability. Crosslinked fibrin can resist thermal degradation, and the quick gelation time of fibrin (as short as 15 s) makes it a good candidate ink for laser-assisted bioprinting [16]. Fibrin bioinks have seen versatile applications in the engineering of various tissues such as skin [56], blood vessels [57], cardiac constructs [58], and so on. The encapsulation of stem cells in fibrin matrices leads to effective gene transduction, while the matrix itself is a good loading vehicle for bioactive molecules [72]. An example [55], is provided below to demonstrate one of its many utilities.

Role of Fibrin in Neural Cell Printing:

An inkjet printer was used to print neural cell (NT2) sheets in a layer-by-layer fashion using fibrin gel [55]. Neurons are anchorage-dependent, and their functionality depends on their attachment onto the scaffolds. Fibrin is a good anchor towards neural cells [73]. Histological staining revealed many NT2 cells entrapped and distributed evenly within the printed neural sheets. The formed fibrin scaffolds exhibited a loose and porous microstructure, which may provide an efficient system to supply nutrients and oxygen to the entrapped NT2 cells [55].

##### Hyaluronic Acid (HA)

Hyaluronic acid (HA), a ubiquitous constituent in the physiological connective tissues, is a linear, non-sulfated glycosaminoglycan composed of alternating units of β−1,4-d-glucuronic acid and β−1,3-*N*-acetyl-D glucosamine residues [16]. It is flexible, biocompatible, biodegradable, and bioresorbable, with a high water content and porosity profile, allowing for the easy diffusion of oxygen, nutrients, and waste products. Due to an abundant negative charge within its structure, HA can absorb large amounts of water and expand its volume up to 1000 times, forming loose hydrated networks that function as a sieve, controlling the transport of water and restricting the movement of pathogens, plasma proteins, and proteases into the matrix network, thereby playing crucial roles as both an immunomodulatory and an anti-inflammatory agent [74]. HA also displays antioxidant effects due to its ability to react with oxygen-derived free radicals [74]. Cell adhesion and proliferation within HA gels are aided by its intrinsic cellular adhesion molecules [72].

Within the water-swollen matrix, crosslinks are infrequent and random, making HA highly soluble at room temperature with a low structural integrity [16]. Hence, HA bioinks usually have functionalized side groups, such as methacrylate, thiol, carbodiimides, hydrides, and tyramine, which are capable of forming covalent bonds to stabilize the polymer. The thiol and methacrylate forms of HA are mostly employed in tissue engineering [16]. Thiolated HA crosslinks occur via air oxidation, and this process can be accelerated by using PEG or gold nanoparticles as the thiol-reactive crosslinking agents. Methacrylate HA forms stable crosslinks by photopolymerization [16]. Bioinks with both low and high viscosities can be formulated using HA since the viscosity of HA solutions increases with an increase in the molecular weight and concentration [75]. A shear-induced reduction in viscosity is also observed, but HA molecules normally require longer relaxation times to reorient themselves. The degradation of HA can take place through enzymes (e.g., hyaluronidase), shear stress, or hydrolysis by acids or bases [75].

Biomedical applications of HA are versatile, such as cartilage engineering [5]. Considering its high expression in tumor cells, and that it is a vital component of the tumor microenvironment, HA bioinks also find their applications for fabrication of tumor models [59]. The following example [5], details one of its many utilities.

Role of Hyaluronic Acid in Osteogenic Induction:

Methacrylated hyaluronic acid (MeHA) (1–3% *w*/*v*) encapsulated with human bone marrow-derived mesenchymal cells (hBMSCs) was bioprinted into 3D constructs for bone-like tissue formation [5]. A total of 0.1% photoinitiator (Irgacure 2959) was used to UV-crosslink the MeHA inks. Various concentrations (1, 1.5, 2, 2.5, and 3% *w*/*v*) of MeHA gels were tested to deduce the optimal concentration that balanced printability with cell viability. UV-crosslinking of MeHA led to more elastic hydrogel than viscous, and the elastic modulus increased with its concentration. Maximum MeHA swelling was seen with a lower concentration (1% *w*/*v*), and the degradation rate matched its swelling profile.

**Figure 6:**
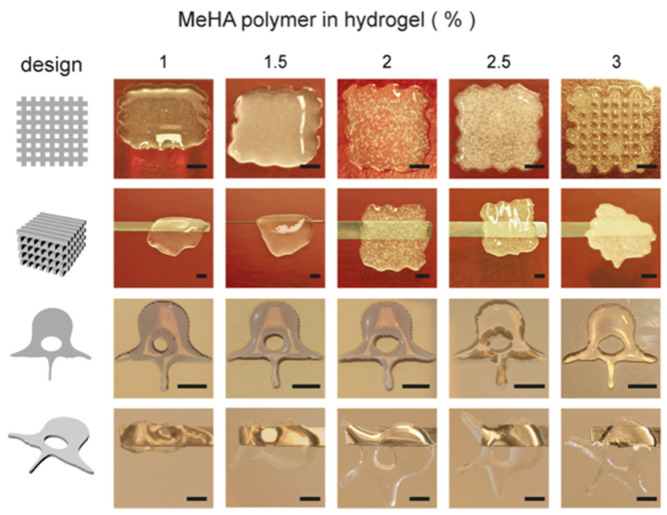
Printability analysis of MeHA at varying concentrations (1 to 3% *w*/*v*) of porous (upper two rows) and non-porous (lower two rows) constructs. Scale bar = 500 μm. Best printing quality was achieved with 3 % *w*/*v* of MeHA. Reproduced from Michelle T. Poldevaart et al. [5].

A sufficient print resolution was achieved with the 3% *w*/*v* MeHA (Figure 6). Cell viability was 73.6 ± 6.4% after 1-day post-printing and dropped to 64.4 ± 12.2% at day 21. Within a lower concentration of MeHA bioinks (1.5–2% *w*/*v*), BMSCs exhibited the elongated morphologies, while 2.5% or above had a much rounder shape.

Osteogenic differentiation of MSCs within the cell-laden constructs was characterized by measuring the calcium (Ca) deposition. Higher concentrations of MeHA (2.5–3% *w*/*v*) increased the Ca deposition, compared to that of the 1.5% *w*/*v* constructs. Mineralization was further increased upon the addition of an external osteogenic stimulus (e.g., bone morphogenic protein 2 (BMP-2)) and 2% *w*/*v* of MeHA constructs showed the highest Ca precipitation, compared to all other groups. Interestingly, the 3% (*w*/*v*) group did not show a noticeable increase of Ca deposition.

##### Alginate

Alginate or alginic acid, an anionic (negatively charged) polysaccharide similar to glycosaminoglycan (GAG) that is found in the native ECM, is refined from brown seaweed. It has two monomeric repeating units: a (1–4)-β-D-mannuronic acid (M block) and an α-L-guluronic acid (G block). The G block helps gel formation, whereas the M block and a combination of M and G blocks increase polymer flexibility [76]. Thus, the mechanical strength and dissolution time of alginate hydrogels are directly related to the G/M ratio within the gel, i.e., a higher G/M content yields a stiffer, slower-dissolving hydrogel [77].

Water and other molecules can be trapped in an alginate matrix via the capillary forces, but diffusion is enabled by the wide distribution of pore size (5–200 μm), a characteristic of alginate bioinks [78]. The amount of crosslinker required to generate a printable hydrogel is 2.5-times greater when using a low-M_W_ alginate compared to a high-M_W_ alginate [78]. The superior properties of alginates include their biocompatibility, low cytotoxicity, fast gelation under physiological conditions, and a slow degradation rate [77]. Alginates show minimal cellular adhesion due to the lack of intrinsic cell adhesive motifs; hence, the gels need to be functionalized with biomimetic peptides such as RGD recognition sites to improve the cell–biomaterial interaction [77].

The gelation of alginates can occur with the presence of divalent ions such as Ca^2+^, Mg^2+^, and Ba^2+^ [17]. Calcium chloride (CaCl_2_), calcium carbonate (CaCO_3_), and calcium sulphate (CaSO_4_) are the most popularly used ionic crosslinkers [78]. The choice of crosslinker and phosphate concentration in the growth medium also has a significant effect on the mechanical properties of the printed constructs [78]. High-molecular-weight alginate solutions crosslinked with CaSO_4_, when prepared in phosphate-containing solutions such as phosphate-buffered saline (PBS) or Dulbecco’s Modified Eagle Medium (DMEM), showed reduced mechanical properties since the phosphate transiently bound to Ca^2+^ ions, thereby affecting the mechanical integrity. Such a phenomenon was not observed with other crosslinkers (e.g., CaCl_2,_ CaCO_3_) in PBS or DMEM [78].

Young’s (storage) modulus is significantly higher in high-M_w_ alginates crosslinked with CaSO_4_, compared to those crosslinked with CaCO_3_ and CaCl_2_. This is mainly due to the solubility of the crosslinkers in an aqueous solution [78]. CaCl_2_ has the highest solubility in aqueous solutions. High solubility leads to rapid gelation and non-uniform crosslinking, both of which can reduce the mechanical properties of the bioink. CaSO_4_ and CaCO_3_ have lower solubility in an aqueous solution; hence, a slower and much more uniform gelation can be established, improving the mechanical properties of the resulting hydrogel [78].

Alginate degradation occurs through the replacement of divalent ions with monovalent ions such as Na^+^. This has a biological significance since alginate polymer chains exceed the filtration size for renal clearance and large displacements of calcium may lead to transient local hypercalcemia [16,79].

In tissue engineering, hollow-filamented alginates have been fused together to form microchannel-like structures mimicking vascular networks [60]. Alginate–poloxamer hybrids were used for cartilage fabrication and alginate–hydroxyapatite–gelatin composites were used in bone printing [61,80]. An exemplar application of pure alginate in the fabrication of an anatomical ear [9], is provided in detail below.

**Figure 7:**
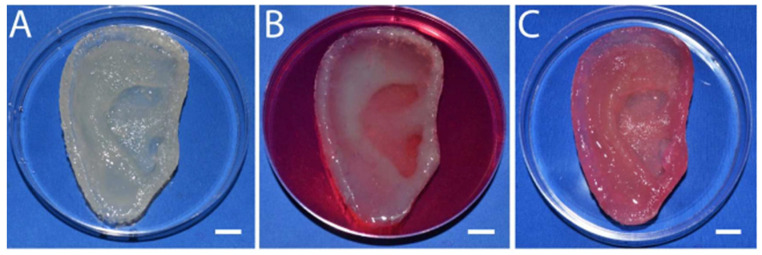
A 3D-printed bionic ear. (**A**) Post-printing. (**B**) Six weeks in culture. (**C**) Ten weeks in culture. Scale bar = 1 cm. Reprinted with permission from [9]. Copyright (2013), American Chemical Society.

3D-Printed Bionic Ear Using Alginate:

Functional electronic components made of nanoelectronic materials were interwoven into a 3D-printed ear auricle from alginate with chondrocytes to create “cyborg organs” [9] (Figure 7). The chondrocyte-seeded alginate hydrogel matrix was extrusion printed with an electrically conductive silver nanoparticle (AgNP)-infused inductive coil antenna connected to the cochlea-shaped electrodes.

Low-viscosity, high-G-content alginate crosslinked with CaSO_4_ was printed with conducting (AgNP-infused) and non-conducting (silicone) solutions, following a CAD drawing of a human ear auricle (Figure 7A). Homologous chondrocyte distribution was achieved, and 91.3 ± 3.9% cell viability was detected post-printing.

Good structural integrity with shape retention was observed, and after four weeks of culture, the construct became increasingly opaque due to the development of new ECM (Figure 7B). At 10 weeks, a good cartilage growth with excellent morphology and tissue level viability was observed (Figure 7C).

The Young’s modulus of the print was dependent on the chondrocyte density, and a lower chondrocyte density resulted in a lower modulus. The hardness across the printed ear was relatively uniform, ranging from 38.50 kPa to 46.80 kPa.

Electrical characterization was performed to observe the ability of the bionic ear to receive signals within and beyond normal audible signal frequencies (20 Hz to 20 kHz). The cochlear-shaped electrode was able to transmit signals across a frequency band of 1 MHz to 5 GHz, and the lobes also showed the excellent reproducibility of audio signals.

##### Agarose

Agarose is a natural and linear polysaccharide consisting of d-galactose and 3,6-anhydro-l-galactopyranose. Agarose hydrogels are thermosensitive and are insoluble in cold water and soluble in boiling water. An agarose solution forms hydrogels when cooled below its gelation temperature of 31 to 36 °C [30]. These hydrogels have time-dependent mechanical properties, leading to stress relaxation similar to the native tissue [72]. The agarose microstructure enables the diffusion of oxygen and other cell-essential compounds. It also exhibits good mechanical toughness, tunable viscosity, and shear-thinning behavior [48]. Agarose is not cell-friendly, with low cell adhesion and spreading. Conjugation with cell adhesive motifs or combining with other bioactive polymers can increase its cell viability [81]. The difficulty of obtaining printable agarose solutions limits its applications for biofabrication; thus, agarose is mainly used as a cell-supporting hydrogel (submerged printing) or as sacrificial molds to create the vasculature within printed constructs [16,33,82]. A possible agarose usage in bioprinting is provided below [29].

3D Model of an Arterial Bifurcation Trunk Using Agarose:

Perfluorocarbons (PFCs) with a high buoyant density were used as the submerged supporting matrix to the extrusion print of 3% wt agarose hydrogels to form vascular bifurcations of arteries. The hydrophobic PFCs increased the contact angle and, therefore, reduced the flattening of the agarose droplets within the PFCs. As a result, the print resolution was improved with a smaller diameter. The minimum droplet size was ∼100 nL.

**Figure 8:**
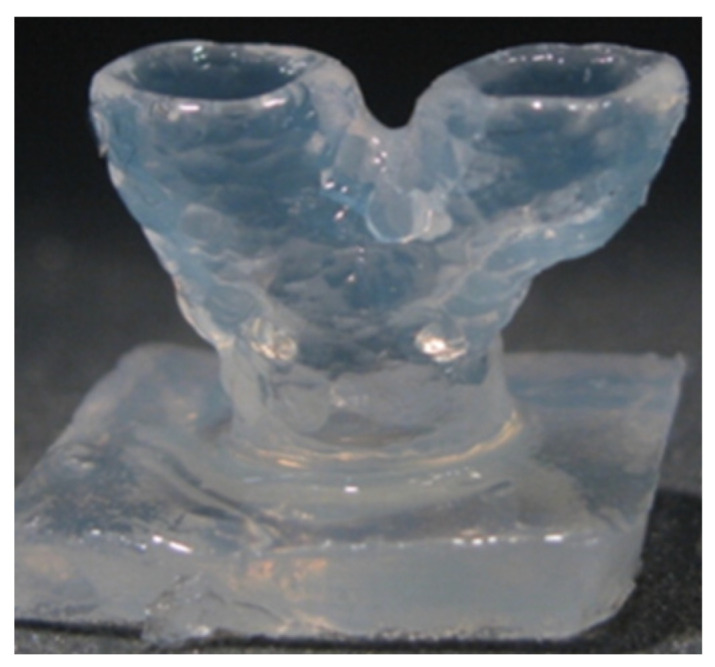
Self-standing arterial bifurcation model. Reproduced from Andreas Blaeser et al. [29].

A 3D model of vascular bifurcation with a trunk and two branches was submerged-printed from the agarose hydrogel [29]. The trunk diameter was 6.3 mm, and the branches were 6.1 mm in diameter (Figure 8). The construct had a wall thickness of ~1 mm with a total height of 10 mm, and a width of 14 mm bifurcation, at an angle of 80°. Although the construct was fabricated from the agarose gel, no solid support was required beneath the branching part or within its lumen.

##### Dextran

Dextran is a hydrophilic, biodegradable, and non-toxic polysaccharide derived from lactic acid bacteria. It is largely composed of linear α-1,6-linked D-glucopyranose residues and can be synthesized from sucrose and maltodextrin as well [83]. The presence of numerous chemically reactive hydroxyl groups enables the formation of interactions with other molecules, thereby reinforcing its structure, and its backbone can be chemically modified to support cell binding [72]. It undergoes biological degradation on its own because of the presence of dextranase within cells [83]. Due to the poor mechanical properties of dextran, it often needs to be combined with other biomaterials to improve its structural integrity. Oxidized dextran can act as a natural crosslinker for gelatin-based bioinks [84]. The incorporation of dextran into bioinks helps to promote tissue vascularization, and the dextran hydrogels have been shown to play crucial roles in wound healing and cartilage development for their ability to enhance neutrophil infiltration [83]. Neutrophils increase the digestion rate of hydrogels, leading to vascular infiltration within the matrix [85]. Dextran also increases cell proliferation and up-regulates gene expressions of endothelial markers [86].

##### Chitosan

Chitosan is a linear cationic polysaccharide obtained by partial de-acetylation of the chitin from exoskeleton of crustaceans, fungi, and insects [72,87]. It is composed of glucosamines (*N*-glucosamine and *N*-acetyl-D-glucosamine), which can be broken down to produce GAGs that make up the ECM [87]. Chitin has a rigid structure with high levels of acetylated groups that diminish its solubility under the physiological pH. Being a weak base (pKa 6.5), it can be dissolved in dilute acids, becoming soluble at a pH of 6.5 or lower [87]. Its de-acylation to chitosan improves its solubility, biocompatibility, and biodegradability (via enzymes such as lysozyme), and its degradation products are non-toxic and non-immunogenic [84].

Chitosan is also bio-adhesive, bacteriostatic (i.e., inhibits bacterial growth), and can act as an antioxidant, chelating, and hemostatic agent. Chitosan hydrogel formation occurs through covalent linking of the chitosan monomers. Crosslinkers such as glutaraldehyde and other reagents such as genipin, palladium cation, di-iso-cyanate, and acrylic acid can also be employed along with photo-initiators to induce the photopolymerization [72,87]. The modification of chitosan with ethylene di-amine tetra acetic acid (EDTA) before the addition of Ca^2+^ enhances its stability and mechanical properties [84].

The formulation of bioinks with chitosan or modified chitosan would yield low toxicity and good printability with tunable mechanical and viscoelastic properties. Concentrations of chitosan ink higher than 11% wt or lower than 4% wt were either too viscous or too diluted, not suitable for extrusion printing [88]. Bioinks with chitosan or modified chitosan were found to provide good support for chondrocyte growth [62]. Chitosan-based hydrogels are also investigated for applications in drug delivery [63] and wound repair [64].

##### Cellulose

Cellulose, the most abundant of the natural polysaccharides, is made of a linear chain of β (1 → 4)-linked D-glucose unit. For 3D printing, nano-fiber celluloses (NFCs), isolated from a plant or bacterial source, are widely used for their improved water retention and gelling capability [89]. Such improvements are mainly from the high surface area of NFCs, which take up water and form a strong hydrogen bond between water and hydroxyl groups of cellulose. Hydroxyl groups of the cellulose can be further modified to incorporate other desirable properties for bioprinting [89].

Cellulose bioink formulation begins with dissolution in alkali solvents such as NaOH/urea, NaOH/thiourea, and ionic liquids to increase the solubility, followed by crosslinking through physical treatments (freeze–thawing, gamma radiation), chemical agents (citric acid, glutaraldehyde), or light irradiation (UV) [90]. Cellulose is primarily degraded by bacteria, fungi, or cellulase [91], and ionizing radiation can enhance the degradation rate by reducing the crystallinity and their molecular weight [92]. Interestingly, cellulose generally does not support bacterial growth, while its incorporation within bioinks could improve cell viability [89]. For example, skin grafts made from bacterial nanocellulose promoted wound healing in [93].

Carboxymethyl cellulose (CMC), a water-soluble cellulose ether, can be used to modulate the viscosity of other polymers for better rheologic performance [84]. In addition, the combination of CMC with other synthetic polymers also favored the cells included in the ink, as CMC provided cell-adhesion sites. Considering that cellulose nanocrystals can promote mechanical strength along with shear-thinning behavior, their incorporation into different bioinks could improve the elasticity, strength, porosity, and integrity of the constructs created [90].

##### Silk Fibroin (SF)

Silk proteins obtained from natural sources such as silkworms and spiders have a fibrous protein core—the fibroin (75%)—along with glue proteins called sericins (25%), which envelop the core protein fibers and play key roles in promoting cell adhesion and proliferation [94]. In addition to water retention, the hydrophilic sericins provide antioxidation, UV resistance, and anti-bacterial properties. Different from sericins, silk fibroin is a block copolymer consisting of both hydrophobic (crystalline) and hydrophilic (amorphous) residues, enabling extensive physical interactions and thereby helping the protein maintain unique mechanical properties such as shear thinning, high elasticity, light weight, high strength, and toughness, coupled with high extensibility, good compressibility, and slow degradation [94,95]. The lack of covalent linkages between silk fibers (showing thermal stability up to 200 °C) also allows for readily processing over a wide temperature range in alkaline conditions [96].

Silk is hygroscopic (moisture retention); thus, under normal conditions (i.e., 20 °C and 65% relative humidity (RH)), it can absorb up to 11% of its weight in water, causing the fibers to swell [96]. Because of its amphoteric nature, the chemical reactivity of silk is rather high, allowing for functionalization to improve its durability [96,97].

The gelation of silk typically results from the formation of intra- and inter-molecular bonds, such as ionic and hydrogen bonding between protein chains. Silk bioinks can be obtained by physical (heating, sonication, photocrosslinking), chemical (solvent treatment), and enzyme-induced methods [98,99,100]. Some changes to the conditions such as temperature increases, fibroin concentration increases, the addition of Ca^2+^, and lowering the pH can promote the interactions between silk fibroin chains and thereby reduce the gelation time [100]. However, silk fibroins themselves do not have cell-binding domains, which need to be incorporated for improved cell adhesion [72]. The exemplar use of silk for fabricating 3D models is detailed below [65].

3D Tracheal Cartilaginous Ring Fabrication Using Silk Fibroin:

A total of 30% SF, modified with glycidyl methacrylate (GMA), was evaluated for the printability and the long-term cell viability of chondrocytes printed in the shape of tracheal rings (7 mm external diameter, 5 mm internal diameter, and 6 mm height) with light-assisted printing (digital light processing—DLP) [65]. Crosslinking was carried out using LAP with a UV light intensity of 3.5 mW cm^−2^.

By varying the concentration (10 to 30%) of Sil-MA, both the compressive and tensile moduli of the Sil-MA gels were evaluated. As observed, the moduli increased with the increase of the Sil-MA concentration. The 30% Sil-MA had the highest modulus (90 kPa) and was able to bear weights of up to 7 Kg and return to its original shape without deformation.

**Figure 9:**
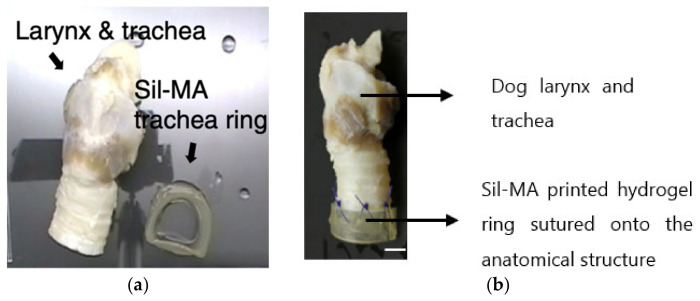
Sil-MA tracheal hydrogel ring. (**a**) Dog larynx and bioprinted Sil-MA hydrogel ring. (**b**) Sil-MA hydrogel tracheal ring attached to dog larynx via end-to-end anastomosis. Scale bar = 1 cm. Reproduced from Soon Hee Kim et al. [65].

The ring-like cartilaginous trachea print was assessed for cartilage formation over a 4-week period post-printing (Figure 9). An even cell distribution was observed throughout the cultured construct, and the cells exhibited round morphologies with lacunae embedded in the basophilic extracellular matrix. The ring structure exhibited sufficient mechanical strength to be attached end-to-end with a dog larynx without structural destruction (Figure 9b). Cell-loaded Sil-MA hydrogel degraded gradually with approximately 50% degradation 4 weeks after cultivation.

##### Gelatin

Gelatin, a denatured and partially hydrolyzed form of collagen, is typically obtained from the bones, tendons, and skin of animals via acidic hydrolysis [16]. It is biocompatible with inherent cell adhesion motifs. Gelatin is thermosensitive, i.e., it forms helical gel-like structures at low temperatures (around 4 °C), and then reverts to a random coil with increased temperature. The low temperature stabilizes the molecule’s tertiary structure, allowing for the formation of physical interactions, thereby resulting in gelation [16]. Quick gelation at the moderate temperatures assures the strong initial stability to the printed constructs, even when printed with other less-stable materials. Meanwhile, bioactive components such as drugs can be entrapped within the gelatin matrix to protect them from cellular oxidation or degradation [101].

While the thermal properties of gelatin make it an ideal candidate for printing, its low melting point (below natural body temperature, i.e., 27 to 33 °C) renders it unsuitable for in vivo applications. In this regard, gelatin has been often used as a sacrificial bioink to form voids or channels within the printed structure upon easy dissolution within an aqueous medium at 37 °C [16]. For in vivo implementation, gelatin typically needs to be chemically functionalized. The amphipathic nature (containing both hydrophilic and hydrophobic side chains) of gelatin makes it capable of forming chemical hydrogels in the presence of crosslinkers [101]. Glutaraldehyde and methacrylamide are the popular choice of crosslinkers, followed by photopolymerization [101].

Considering the high viscous nature of crosslinked or semi-crosslinked gelatin bioink at room temperature, extrusion printing is predominantly adopted. The 3D printing of gelatin usually occurs by either extruding the warm unmodified gelatin solution onto a cold stage to induce gelation or directly extruding the cold, fully crosslinked gel. The former produces structures with a poor resolution due to the filament spreading, while the latter leads to clumpy, inhomogeneous strands with irregular pores [16]. Ideal printing of gelatin can be accomplished by partial crosslinking in combination with shear-thinning. In bioprinting, gelatin can function as: (i) stand-alone structures providing mechanical support and biological cues through chemical crosslinking, (ii) sacrificial materials enabling vascularization within bioprinted constructs, or (iii) thermo-reversible supports during the printing process by acting as a support bath for bioink deposition [16]. An example highlighting the possibilities of using gelatin in bioprinting is detailed below [66].

**Figure 10:**
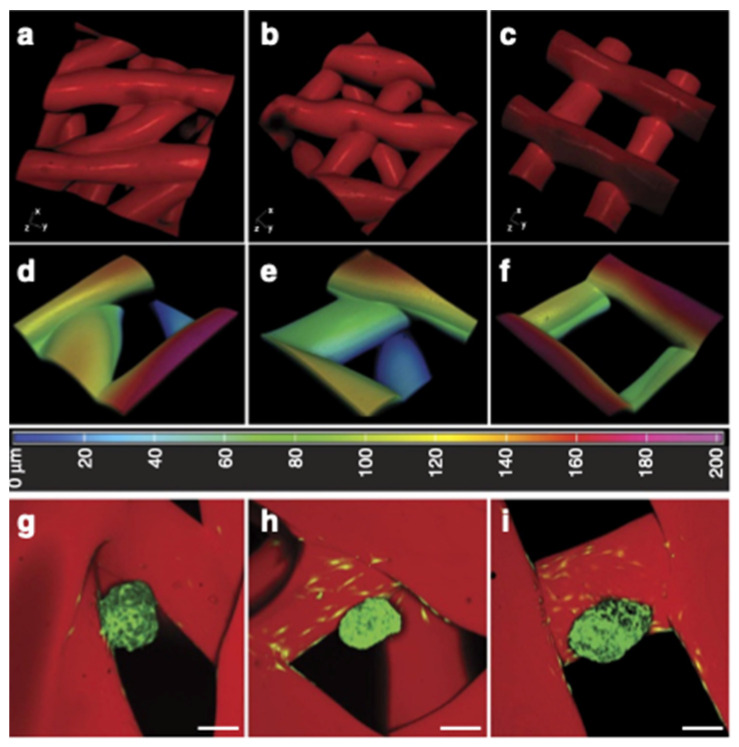
Three-dimensional reconstructive confocal fluorescence images of follicle–strut contact geometries. (**a**–**c**) 30° (**a**,**d**,**g**), 60° (**b**,**e**,**h**), and 90° (**c**,**f**,**i**) angled scaffolds. (**d**–**f**) Pore depth reconstruction; colors correspond to pore depth. (**g**–**i**) Follicles seeded onto scaffolds (2-day culture image). Follicles in 30° and 60° pores tended to reside in the corners, whereas follicles in 90° pores were more likely to be along only one strut. Scale bar = 100 μm. Reproduced from Monica M. Laronda et al. [66].

Bioprosthetic Ovarian Constructs Using Gelatin:

Gelatin hydrogels (10% *w*/*v*) and murine ovarian follicles were used to create bioprosthetic ovaries [66]. A smooth and continuous filament was obtained by printing slightly crosslinked gelatin at 30 °C. The partially crosslinked gel had a weaker storage modulus with a higher strain and a lower critical stress, which was weak enough to be extruded while retaining its shape. A total of five layers with dimensions of 2 cm (W) × 5 cm (L) × 0.5 cm (H) were extruded on a cooled stage (10 °C). To better stabilize the printed structure, further crosslinking with *N*-(3-dimethyl aminopropyl)-*N*-ethyl carbodiimide (EDC)/N-hydroxysuccinimide (NHS) was conducted after printing. During printing, the overlay angles of consecutive layers of filaments could be tuned to yield various structures. In this study, three overlay angles (30°, 60° and 90°) were selected (see Figure 10a–f). A culture of ovarian follicles onto the printed scaffolds revealed that the 90° scaffolds did not better support the survival of follicles upon extended culture (after 8 days, 48.47 ± 8.31% died) in comparison to 30° and 60° scaffolds with a higher survival rate (i.e., 78.57 ± 3.57%, 75.89 ± 4.04%, respectively), which might be due to the limited physical confinement to follicles and cellular dissociation (Figure 10g–i). Experimental results implied a positive correlation between the follicle survival and the number of strut contacts. Follicles within the 30° and 60° scaffolds had an increased chance of contacting two or more struts, while in the 90° scaffolds, they had only a limited contact. The 30° and 60° scaffolds were able to support in vitro follicular differentiation, i.e., hormone production, oocyte maturation, and ovulation. The 60° scaffolds were able to provide larger pores for better follicle seeding throughout the entire depth of the scaffold.

The follicles cultured on the 60° scaffolds for 4 days were implanted in adult mice with both ovaries removed surgically. Within the first week implantation, the bioprosthetic ovaries became vascularized without the presence of exogenous angiogenic factors. The implanted bioprosthetic ovaries were able to support fertilization once grown to adulthood, and the mice sired or gave birth to their litters.

##### GelMA

The crosslinking of gelatin with glutaraldehyde is no longer extensively practiced due to the foreseeable toxicity from glutaraldehyde. Thus, the current attempts for in vivo uses of gelatin are made mainly through its methacrylated form—GelMA [16]. GelMA does not require crosslinking agents or localized gelation; instead, the methacryolyl substitutes enable gelatin to be photocrosslinked in the presence of light and a photoinitiator. The functional amino acid domains of gelatin such as cell adhesion (R-G-D) motifs and matrix metalloprotease (MMP)-degradable motifs remain intact during its methacrylation; hence, GelMA can retain the cell adhesive and biodegradability properties of gelatin well [102].

GelMA synthesis occurs by the direct reaction of gelatin with methacrylic anhydride (MA) in PBS at 50 °C and physiological pH (7.4). By varying the amount of MA added to the reaction mixture, physical properties of GelMA can be tuned. The reaction is stopped by diluting with phosphate buffer, followed by dialyzing with deionized water to remove low-molecular-weight impurities [103]. The maintenance of physiological pH is essential, as the methacrylic acid by-product during the reaction can reduce the isoelectric point (pH at which a molecule carries no net charge) of gelatin, causing a reduction in its amine-containing residues necessary for the reaction [103]. Low concentrations of GelMA (3% *w*/*v*) allow for the production of cell-laden constructs with a high shape fidelity. The GelMA bioink has self-healing (at concentrations of 3% and 4%) and shear-thinning properties [102]. The use of GelMA for nerve conduits is detailed below to further elaborate its distinct properties [67].

**Figure 11:**
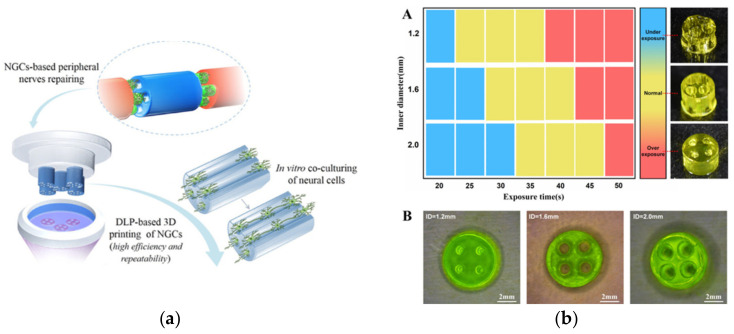
Nerve guidance conduits from GelMA. (**a**) Schematic illustration of 3D-printed NGCs. (**b**) Fabrication of GelMA-based NGCs. (**A**) Photograph of photocrosslinked of channel conduits at different exposure times. (**B**) Photograph of NGCs with varying internal diameters (IDs). Scale bar = 2 mm. Reproduced from Wensong Ye et al. [67].

GelMA-Based Nerve Guidance Conduits for Peripheral Nerve Injury:

Cylindrical 4-channel nerve guidance conduits (NGCs) with lengths of 5 mm and outer diameters of 6.0 mm, but with varying internal diameters (1.2, 1.6, and 2 mm), were fabricated from GelMA (13.3% *w*/*v*) using digital light printing (DLP), a subsection of stereolithography [67]. Crosslinking was carried out using 0.25% *w*/*v* of LAP with visible light (405 nm) for 35 s (Figure 11). Crosslinking < 20 s was too feeble to produce mechanically stable structures, and >50 s yielded overcured structures with blocked channels at the bottom. The compression strength increased with a decrease in internal diameter.

The printed NGCs were first tested for their supportiveness to neural cells by culturing with PC-12 cells (a pheochromocytoma cell line). All the printed NGCs supported initial cell adhesion with a high cell viability (>95%) on day 1. On day 7, homogenous cell distribution with extended cytoskeleton morphology and evidence of proliferation was confirmed. The PC-12 cells migrated deep into the conduit, following the longitudinal channel path, resembling longitudinally aligned neural cell strands that guide axon regeneration. NGCs with a larger inner diameter significantly increased cell migration over time compared to those with a smaller inner diameter, as the reduced internal diameter led to cell crowding, inhibited cell migration, and limited nutrient exchange.

Neuronal crest stem cells are multipotent and can give rise to peripheral neurons. Their neuronal differentiation on the NGCs was also evaluated. After 10 days of culture, an early neuron-specific marker and a neuron axon-specific marker were both detected, indicating the ability of NGCs to induce neuron differentiation in vitro.

##### Matrigel

Matrigel is the trade name of the reconstituted basement membrane proteins and small molecules primarily composed of collagen type IV, laminin, perlecan, and other growth factors secreted by Engelbreth–Holm–Swarm mouse sarcoma cells. Matrigel is typically solubilized in a serum-free solution and is kept refrigerated (4 °C) until use. Gelation occurs at 37 °C and solidifies approximately within 30 min [104]. The rapid thermal gelation makes the extrusion printing of Matrigel very difficult; hence, inkjet or laser-based printers are adopted to form Matrigel droplets on the substrate [10].

Considering the origin of Matrigel from tumor cells and its inconsistent and poorly defined composition, its use is generally limited to in vitro cultures instead of in vivo human applications [104]. Cultures of cells in or on Matrigel often induces various differentiations, depending on the cell type. Malignant and normal cells exhibit different activities with Matrigel, e.g., normal fibroblasts were seen to form small non-invasive colonies, while fibrosarcoma cells (HT1080) rapidly invaded into the gel to form multiple tunnels with a high proliferation rate [104].

The close compositional resemblance of Matrigel to the basement membrane makes it an ideal choice to replicate the basement membrane–cell interactions, making it an optimal matrix for evaluating angiogenesis, adipogenesis, and cell differentiation, and for culturing stem cells with self-renewal pluripotency [16,104]. As a supporting matrix, Matrigel enabled the printed droplets of ovarian cancer cells and fibroblasts to develop a more biomimetic 3D coculture system for the in vitro study of ovarian cancer [16,68].

#### 3.1.2. Synthetic Bioinks

While natural bioinks are more favorable for emulating the cellular microenvironment, synthetic bioinks have been demonstrated to be more adaptable and versatile. They are more chemically defined, with tunable mechanical and rheological properties, controllable degradation rates, and batch-to-batch consistency. Meanwhile, the ability to include desirable chemical manipulations, such as the addition of crosslinking sites and biomimetic molecules, is also beneficial. Synthetic bioinks are typically bio-inert; thus, the incorporation of cell adhesive molecules is necessary to induce the preferred cell–biomaterial interactions. Representative synthetic bioinks from Table 3 are elaborated in detail below, along with exemplar applications.

##### Polyethylene Glycol (PEG)

Polyethylene glycol (PEG) is a linear hydrophilic polyether, synthesized from ethylene oxide. Based on the levels of polymerization, it can be termed PEG (molecular weight (M_w_) < 20 kDa), polyethylene oxide (PEO: M_w_ > 20 kDa), and poly oxyethylene (POE: any M_w_). These distinctions help create PEG polymers with tailorable rheological and mechanical properties by simply adjusting the molecular weight [16]. Because of the high water-solubility and strong mechanical properties, PEG can maintain its shape during and after printing. On the other hand, the mobile, non-ionic, and highly hydrating nature of PEG makes it resistant to unwanted molecular adhesion, thereby enhancing its non-immunogenic property [15].

Both physical and chemical gelation approaches can be used to form PEG hydrogels, but chemical crosslinking would allow the introduction of degradable crosslinkers and the tuning of physiochemical properties (e.g., permeability water content, elastic modulus, etc.) [17]. PEG crosslinking can be achieved through chain-growth, step-growth, mixed-mode (hybrid of chain and step), and photopolymerization [15,16].

Loosely crosslinked PEG hydrogels have high water content (>95% of mass), resembling soft tissue and facilitating easy nutrient–waste exchanges. The increase in permeability can be detrimental to encapsulated cells, as it cannot prevent smaller cytotoxic molecules (cytokines, reactive oxygen species) from passing through the hydrogel barrier and triggering cellular apoptosis, which eventually leads to cell viability loss [15]. The stealth or anti-fouling property of PEG helps alleviate this shortcoming by repelling non-specific protein adsorption and cell adhesion, thereby reducing the adhesion of inflammatory cells onto the hydrogel surface to decrease fibrotic capsule formation. However, this property also hinders the adsorption of bioactive molecules, such as extracellular matrix (ECM) proteins, that support the growth and function of the encapsulated cells; hence, they can reduce the viability of the residing cells for the lack of interactions with a surrounding matrix [15]. The inclusion of cell-binding moieties within the PEG structure is a good strategy to overcome this shortcoming [15,16]. Common PEG derivatives, such as PEG-norbornene (PEGNb), PEG monoacrylate (PEGA), PEG diacrylate (PEGDA), PEG methacrylate (PEGMA), and PEG dimethacrylate (PEGDMA), are also good candidates for synthetic bioinks [16]. An example of using PEGDA for 3D model fabrication [69], is detailed below.

**Figure 12:**
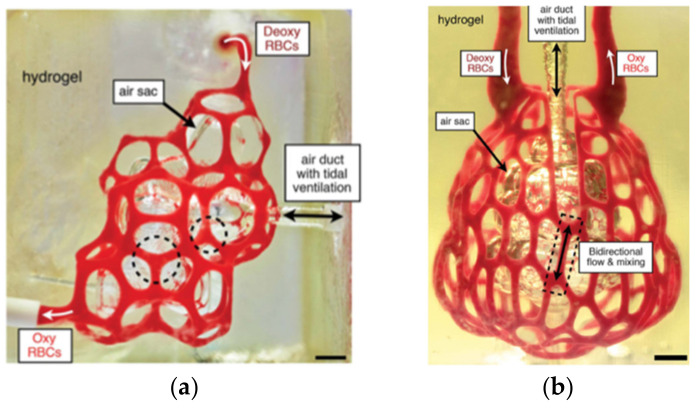
PEGDA-assisted development of alveolar and lung models. (**a**) Photograph of printed alveolar model during RBC perfusion, while air sac was ventilated with oxygen; dotted circles represent RBC vessel compression. (**b**) Photograph of printed distal lung sub-unit during RBC perfusion, while air sac was ventilated with oxygen. Scale bar = 1 mm. Printed with permission from [69]. Copyright (2019), *Science*.

Alveolar Model Using PEG:

PEGDA (20% wt, 6 kDa) was used together with tartazine, a synthetic light-induced crosslinker, to generate hydrogels following the thiolene step-growth polymerization. The PEGDA hydrogel was first printed using stereolithography. A 3D alveolar model was initially established and perfused with red blood cells (RBCs) to test the feasibility of mimicking airway and RBC exchange in the lung [69]. Upon airway inflation with humidified oxygen, the concave regions of the model airways squeezed adjacent blood vessels and caused RBC clearance. The compression of RBC vessels acted as switching valves to redirect the fluid streams to neighboring vessel segments (Figure 12a). The alveolar model was extended to a scalable lung-mimetic design with a branched airway. Inlet and outlet blood vessels were grown 180° opposite to each other, and the tip of the lung unit was populated with alveolar cells (Figure 12b). It was revealed that the PEGDA hydrogels could withstand more than 10,000 ventilation cycles for over 6 h of RBC perfusion.

##### Poloxamers

Poloxamers, trademarked as Pluronic^®^ and Lutrol^®^, are amphipathic triblock copolymers with a base molecular structure of polyethylene oxide–polypropylene oxide–polyethylene oxide (PEO–PPO–PEO) [16]. The reduced solubility of PPO in the temperature range of 22 to 37 °C makes poloxamers inverse-thermosensitive, i.e., it is a solution below 22 °C and is gel-like above 37 °C [16,19]. Gelation is dependent on the ratio between PPO and PEO and the total polymer length. The self-assembled structures can be stabilized via photopolymerization by including photocrosslinkable side groups, such as acrylates. Pluronic is soluble in aqueous solutions with an in vitro degradation time of ~1 week [16].

The non-polar side groups of Pluronic closely resembles phospholipids; therefore, its cytocompatibility with the cell membrane is reduced. This, combined with the lack of intrinsic cell adhesion domains, results in a cell viability between 4 and 60% [16]. The bio-inertness, coupled with thermo-responsive gelation, makes poloxamers the ideal sacrificial templates [19]. Due to its high viscosity, the principal advantage of Pluronic is the possibility of creating accurate structures with good shape fidelity immediately after printing [16].

##### Poly Vinyl Alcohol (PVA)

Poly vinyl alcohol (PVA) is a water-soluble, biodegradable, and biocompatible polymer synthesized from vinyl acrylate. It is chemically inert in numerous organic solvents and exhibits optical transparency in the UV–visible region, and the non-modified PVA has anti-fouling properties [105]. Its bio-inert characteristics are mainly from its strong hydrophilicity, inhibiting water circulation to cells and limiting cells from adhering to its surface [80].

PVA hydrogel synthesis typically involves repeated cycles of freeze–thawing. A homologous solution of PVA and dimethyl sulphoxide (DMSO) is frozen at −20 °C for 10 h in the presence of nitrogen atmosphere, followed by thawing at 25 °C for 2 h [106]. The incorporation of DMSO into water as a solvent improves PVA transparency. The freeze–thaw cycling produces PVA hydrogels with high mechanical properties, high water content, and excellent transparency [105]. PVA gels can also be produced by cooling the mixed solvent to room temperature without the need of freezing. In this way, the solvent (DMSO) can be removed by immersion in de-ionized water [105]. The hydrogel crosslinks can be reinforced with chemical agents or radiation [80].

The increased number of hydrophilic groups (-OH) within PVA enables it to form hydrogen bonding with free water molecules so that water and other small molecules can easily penetrate the polymer network. The degree of swelling declines with an increase in concentration or crosslinking [106]. At a high water content, the viscoelasticity of PVA is similar to that of natural tissues such as articular cartilages, and at low water contents, an elastic response dominates. Hence, PVA hydrogels have found biomimetic applications in orthopedics and artificial vascular replacements [80].

##### Silicone

Silicone polymers were initially manufactured into 3D models using casting, injection molding, or compression molding, since the extreme heat resistance of silicone prevented it from being additively manufactured in a layer-by-layer fashion [107]. The development of a novel photopolymerizable extrusion platform enabled silicone to be rapidly extruded from a printer nozzle [71]. These polymers have the versatility of being fabricated into elastomers, adhesives, or gels, based on their intended applications, ranging from acting as coatings on medical devices, to implants (mammary prosthetics) and physiological 3D models [107]. This highly biocompatible, elastic, and hydrophobic polymer exhibits UV, heat, and chemical resistance while maintaining toughness, gas permeability, and transparency [107]. In [71], 3D-bioprinted silicone meniscus implants exhibited strain hardening and hysteresis, and in the presence of fibroblasts, the silicone models exhibited good biocompatibility.

#### 3.1.3. Composite Bioinks

A composite is a multi-component material comprised of different phase domains in which at least one type of phase domain is a continuous phase [108]. Single-component hydrogels formed from natural or synthetic polymers are faced with drawbacks such as mechanical insufficiency and biocompatibility. To properly emulate the anisotropic nature of intrinsic tissue structures and their complex microenvironments, composite bioinks from mixtures of distinct constituents with different chemical, physical, and biological properties are often employed.

In vivo, the ECM itself is a composite, with its water-swollen hydrogel-like 3D structure as the continuous phase and the dispersed phase represented by particles and fibrous structures of organic or inorganic origin. Natural bioinks can be combined with structurally robust biomaterials, usually of synthetic origin, to improve their structural integrity and printability, while synthetic materials are coupled with their natural counterparts to improve bioactivity. In addition to biomaterials, nanoparticles (e.g., gold, silver, nanosilicates, iron oxide), minerals (e.g., hydroxyapatite), and/or bioactive molecules (e.g., growth factors, cytokines, peptide sequences) can also be formulated in the bioink to form composites (Table 4). A few 3D-bioprinted hybrid models studies have been detailed below.

##### Collagen–Gold Nanowires in Muscle-Tissue Engineering

Collagen (5% wt) amalgamated with gold nanowires (GNWs—diameter 30 nm, length 4500 nm) was used to achieve accelerated cell alignment and biomimicry of the electrical properties of muscle tissue (C2C12, myoblast cell lines) during cell printing [109]. The positioning of the GNWs was accomplished by applying electric stress through a micro-sized nozzle and an electric field (5V, 1Hz) post-printing, as the electrostatic force can enhance myosin synthesis and the formation of functional fibers. The capability of supporting-cell remodeling and polarization from collagen, in combination with the ability of GNW to deliver topographic cues to the cells, made the cell-laden collagen–GNW bioinks ideal for muscle-tissue regeneration.

**Figure 13:**
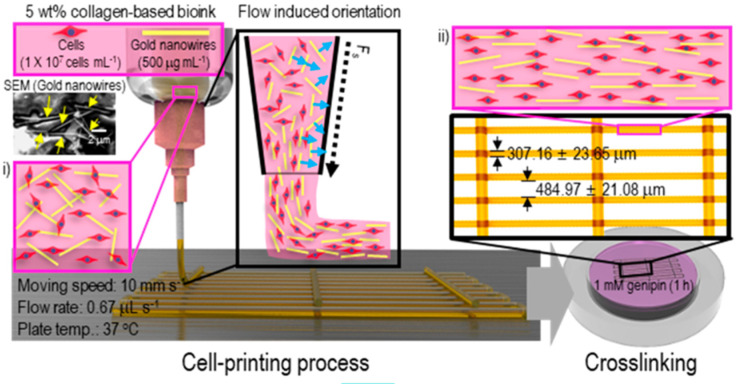
Schematic illustrations of collagen–GNW bioprinting. (**i**) Randomly aligned GNWs—before printing; (**ii**) Aligned GNWs—after printing and crosslinking. Reproduced with permission from [109]. Copyright (2019), American Chemical Society.

The nozzle moving speed and bioink flow rate directly affected the alignment of GNWs within the print. A high nozzle moving speed (15 mm/s) led to a high GNW alignment within the printed bioink struts, but the struts were significantly unstable (non-continuous). A flow rate of 0.67 μL/s and a nozzle speed of 10 mm/s provided highly aligned GNWs with stable struts (Figure 13). Two different processing temperatures (25 °C at the nozzle and 37 °C at the working plate) were used to achieve an optimal print structure with a high cell viability (>90%).

Compared to collagen-alone bioink, bioinks containing GNWs (either random or oriented-GNW distribution) had enhanced Young’s moduli, but clearly had a reduced maximum strain and a low electrical resistance. The bioinks with oriented GNWs were the only ones that showed anisotropic behavior. C212 myoblasts in the oriented GNWs inks showed a similar alignment on days 7 and 14, and no alignments were noticeable in the random GNWs group. As observed, GNWs could serve as cell anchorage sites to promote cell attachment and growth, hence the constructs with GNWs showed a higher cellular proliferation. Muscle tissues typically exhibit asymmetric electrical transmission; thus, the effects of an electric field on the myoblast alignment within the printed constructs were also analyzed. Interestingly, the myoblast alignment was obvious in parallel to the electric field, compared to those subjected to a perpendicular and to no electric field. Cell-laden constructs subjected to a parallel electric field also showed the highest myogenic expression after 14 and 21 days of culture.

##### Agarose–Laponite Nanosilicates in Fibroblast Culturing

Agarose hydrogels have mostly been used as a support structure due to their low shear moduli and their lack of intrinsic cell-binding sites. At higher concentrations, agarose would assemble into stiff helical fiber bundles, causing easy structural breakdown during extrusion at a high shear rate. The incorporation of nanomaterials such as Laponite can improve both the flow behavior and the biological functions of agarose [82].

**Figure 14:**
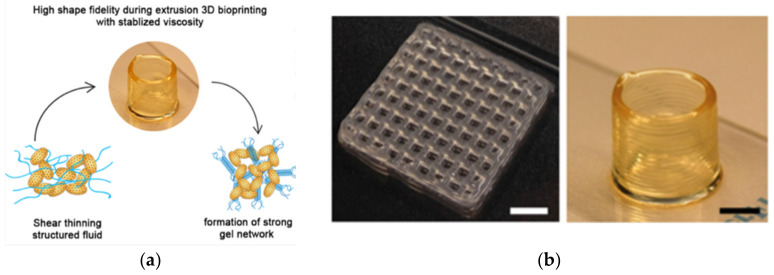
(**a**) Schematic illustration of the fluidity and structural fidelity of the agarose–Laponite bioink. (**b**) The 3D-bioprinted nanocomposite constructs containing 3% wt agarose and 2% wt Laponite. Scale bar = 5 mm. Reproduced with permission from [82]. Copyright (2019), American Chemical Society.

Laponite is a synthetic disc-shaped nanosilicate that possesses a negative surface charge and a positive edge charge when dispersed in water. Such a heterogeneous charge distribution leads to the formation of a structure with thixotropic behavior as a result of inter-nanoparticle ionic interactions that are extremely responsive to shear deformation with a high degree of recoverability (Figure 14a). Thus, Laponite can enhance the shear modulus of agarose over a large range of shear strains.

By varying the concentration of Laponite (1 to 3% wt) in HeLa and NIH-3T3 (mouse embryonic fibroblast)-laden agarose (3% wt) hydrogels, the flowability of the bioink could be tuned, and no gelation was observed at a concentration < 2% wt [82]. A significant increase of the toughness and yielding strength was seen with the increase of the Laponite content, but this did not happen to the compression modulus. The 2% wt Laponite was found to be the optimal concentration for 3D bioprinting. The bioink was extrusion printed with the nozzle temperature at 37 °C and the building platform at room temperature. Different from pure agarose gels, the presence of Laponite did induce some collapse of the printed fiber, forming thicker fibers because of the interaction between agarose and the charged surface of the Laponite. The printed composite constructs containing HeLa and NIH-3T3 maintained their characteristic shape through the entire incubation period of 7 days, and the normalized cell viability was around 100% on days 1, 3, and 7 (Figure 14b). Increased metabolic activity and cell spreading was also observed with NIH-3T3 cells in the presence of Laponite.

##### Alginate–Poloxamer Composites for Cartilage Formation

**Figure 15:**
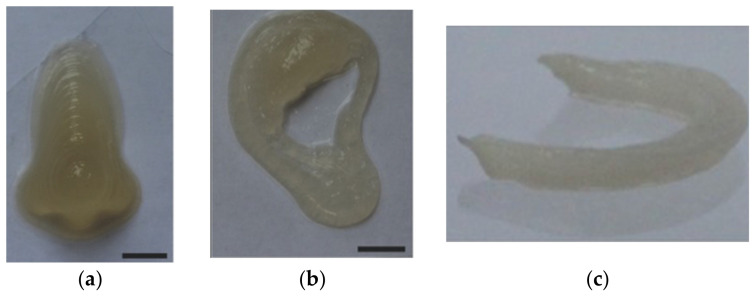
Printed constructs from alginate (6% wt)–Pluronic (13% wt) hybrid gels. (**a**) Non-cell-laden prints of an anatomical nose with a height of 1.72 cm (**b**) Non-cell-laden prints of an anatomical ear with a height of 0.64 cm. Scale bar = 1 cm. (**c**) hMSC-laden full-sized tracheal cartilage ring (17 mm (L) × 14.5mm (W) × 1.8 mm (H)) after culture for 35 days. Reproduced from James P.K Armstrong et al. [111].

Because of its thermo-responsive sol–gel transition in the physiological temperature range, Pluronic (F-127), also called Poloxamer gels, are themselves often used only as fugitive inks for vascular chamber formation [110]. The rapid crosslinking ability of alginate by divalent ions provides excellent structural fidelity in aqueous solutions; however, this attribute also prevents effective interlayer adhesion during layer-by-layer immersion printing. In this regard, the Pluronic–alginate hybrid assemblies provide instant solidification via the sol–gel transition of Pluronic and allow the stabilization of the printed structures via alginate crosslinking upon CaCl_2_ immersion. The Pluronic constituent also serves as a sacrificial template, resulting in the formation of micron-sized pores or anisotropic microchannels within the printed construct. Thus, 3D constructs containing hMSCs were fabricated via the extrusion printing of cell-laden alginate–poloxamer gels [111]. The optimal formulation for printing was the combination of 13% wt F-127 and 6% wt sodium alginate, enabling smooth prints with good geometric reproducibility. The printed structure could retain its geometry for up to 5 days, and the structural integrity loss after 5 days was mainly due to the efflux of Ca^2+^ into the culture medium, leading to osmotic swelling of the printed structure. This could be counteracted by supplying the culture medium with millimolar CaCl_2_. Crosslinking of the hybrid gel with Ca^2+^ resulted in an increased shear modulus, approximately twice that of the crosslinked 6% wt alginate alone. The Young’s modulus (E) of the crosslinked hybrid bioink was 50% higher than that of crosslinked 6% wt alginate, and it was similar to the modulus of soft tissues such as articular hyaline cartilage as measured in bovine tissue. A series of anatomical structures such as a nose and an ear were printed using the hybrid gel without hMSCs (Figure 15a,b).

Cell-encapsulated alginate–F-127 bioinks were printed into a tracheal cartilage ring. F-127 itself does exhibit some cytotoxicity, but not when used as a liquid additive. Hence, the hMSCs were only exposed to 13% wt F-127 during mixing and printing (typically 30–60 min), and afterwards, the F-127 was removed from the hybrid gel. The viability of the encapsulated hMSCs was found to be 87 ± 4% (immediately after printing) and 83 ± 6% (at day 7). The tracheal ring was cultured in the medium supplemented with growth factors known to induce chondrogenesis or osteogenesis. The printed constructs possessed excellent structural fidelity throughout the culture period (35 days), and the differentiated cells retained their capacity for ECM production.

##### PVA–Chitosan for the Fabrication of Bioprinted Cornea

PVA has been extensively used as a carrier in corneal engineering on account of its transparency, elasticity, good oxygen permeability, and mechanical stability. The inclusion of chitosan into PVA could increase the porosity and solvent diffusion. Corneal constructs from PVA (13% wt) and chitosan (1, 3 and 5% wt) were 3D-printed and tested for biocompatibility using human adipose-derived mesenchymal cells (hASCs).

**Figure 16:**
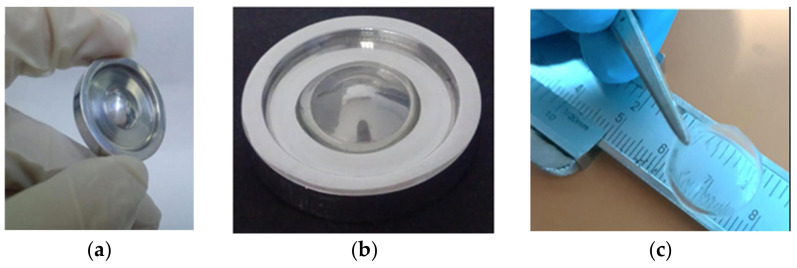
Biofabrication of a corneal construct from PVA and CS composite hydrogels. (**a**) Aluminum mold. (**b**) Mold with corneal construct: post-printing. (**c**) The 3D-bioprinted cornea. Reprinted with permission from [112]. Copyright (2020), Elsevier Ltd.

An aluminum corneal mold was fabricated (Figure 16a) and used to guide the bottom-up printing of a corneal structure for the exact shape (Figure 16b,c) [112].

The thickness of the printed corneal construct (136 µm towards the periphery and 400 µm at the center) was similar to the native corneal stroma (central thickness of ~478 to 500 µm). Swelling rates increased with the increase in chitosan amount. Increased swellability implied a higher surface-to-volume ratio and a higher solute diffusion across the structure, both of which aid cells to attach, grow, and proliferate in the 3D constructs. However, high swelling rates caused the decrease of hydrogel transparency. Since this study aimed to mimic the structure and geometry of human cornea, the higher swellable constructs were crosslinked to a greater degree to avoid the loss of transparency. The transmittance ability of the constructs was tested with the wavelength range between 400–800 nm. Pure PVA had ~61% transmission ratio, while the presence of CS decreased the value to 56%, 53%, and 49% for the 1%, 3%, and 5% CS constructs, respectively. For comparison, the human cornea has a transmittance of 90%. To reduce the loss of transparency, the constructs with higher swellability were, therefore, crosslinked. In addition, adding CS into the PVA also increased the degradation, and the 5% CS had a higher degradation rate compared to others.

The hASCs were able to attach to the surface of the PVA-based corneal stroma constructs with a characteristic fibroblastic morphology, and both pure PVA and CS-containing constructs supported good cell viability over a 7-day culture. Nuclei were seen clearly with prominent nucleoli and secretory granules for all constructs. Fast cell growth and large cell spreading were particularly observed with the 3% CS constructs.

##### Silk–Gelatin Hybrids for Bioprinting Skin

**Figure 17:**
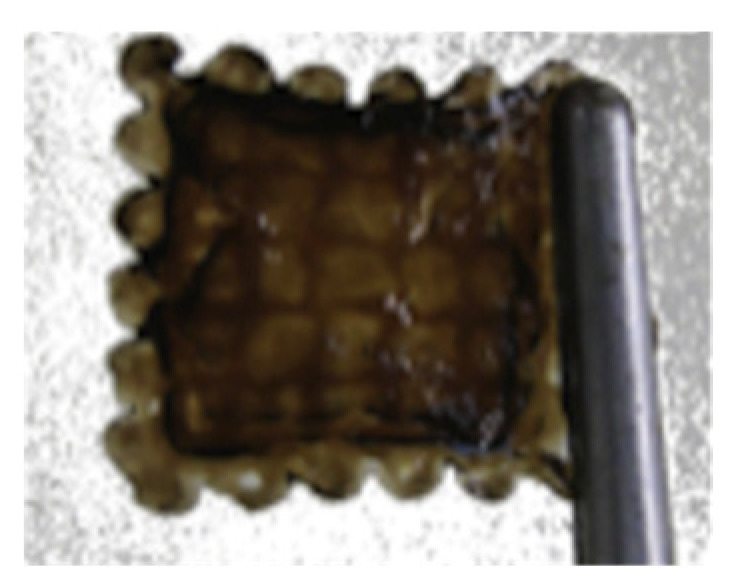
Bioprinted skin. Reprinted with permission from [113]. Copyright (2019), Elsevier Ltd.

The silk–gelatin bioink offers excellent printability and supports long-term cell viability. The silk fibroin could modulate the key signaling pathways responsible for targeted cell differentiation. A multi-layered skin equivalent was printed in a layer-by-layer fashion from the 5% silk and 5% gelatin hybrid bioinks laden with respective human primary fibroblasts and keratinocytes [113]. The structural stability of the printed construct was provided by enzymatic crosslinking with tyrosinase, and the integrity of the printed structure was preserved for up to 4 weeks (Figure 17). No significant changes in the overall dimensions were observed, even after 21 days.

The bioprinted skin equivalents showed a linear response to strain throughout the elongation course and had a much lower tensile modulus (0.03 ± 0.005 MPa) than native skin (5.25 ± 2 MPa). Both fibroblasts and keratinocytes were distributed uniformly throughout the construct, and a 96% cell viability was maintained throughout the 21-day culture. However, the cells experienced morphology changes along with matrix remodeling. Fibroblasts in the dermal layer underwent a transient change, from round to spindle shape, during the first 3 days, while keratinocytes displayed a more elongated morphology at day 21, as compared to those at day 7. Fibroblasts remained embedded in the silk–gelatin matrix during the entire culture period. However, keratinocytes gradually displayed the front–rear polarity to migrate toward the pores within the printed structures, leading to complete coverage of pores by 21 days. The in vitro keratinocyte migration is synonymous with the response of in vivo keratinocytes to skin injury during wound healing.

##### Matrigel–Agarose Composites in Biomimetic Intestinal Model

Agarose has thermo-responsive gelation and proper mechanical properties for 3D printing. However, the lack of cell-binding motifs makes it unsuitable for many tissue cells to attach and spread. Matrigel has been deemed ideal for intestinal stem cell cultures. Hence, the hybrid of agarose and Matrigel was used to establish an in vitro biomimetic intestinal model [114]. Agarose solidified at temperatures below 32 °C, whereas Matrigel gelation occurred at temperatures above 4 °C. Mixing cold Matrigel with hot agarose solution caused the partial gelation of the hydrogel. The addition of Matrigel (15, 30, or 50% *v*/*v*) into a 2 wt% agarose solution affected the elastic property of the hybrid hydrogel. The addition of 15% and 30% Matrigel could yield sufficient mechanical properties (G′ (elastic modulus) > 1800 Pa) for printing, whereas the addition of 50% Matrigel (G′~850 Pa) failed to maintain the structural fidelity. However, a higher Matrigel fraction (e.g., 50%) favored the spreading of human colonial epithelial cells (HCT116) (37%). In consideration of the lowest storage modulus with 50% Matrigel, increasing the concentration of agarose stock solution from 2% to 3% wt was able to increase G′ to 1250 Pa for printing. A majority (72%) of HCT116 cells in the agarose–Matrigel hybrid hydrogel showed spindle-like shapes and the number of cells increased by day 8. However, between day 8 and day 11, the percentage of spread cells decreased from 70% to 47%. Cell viability remained relatively high within the first six days (77%) but dropped after seven days due to a deficit in nutrient supply.

##### Cellulose–Carbon Nanotubes for In Vitro Neural Growth Models

**Figure 18:**
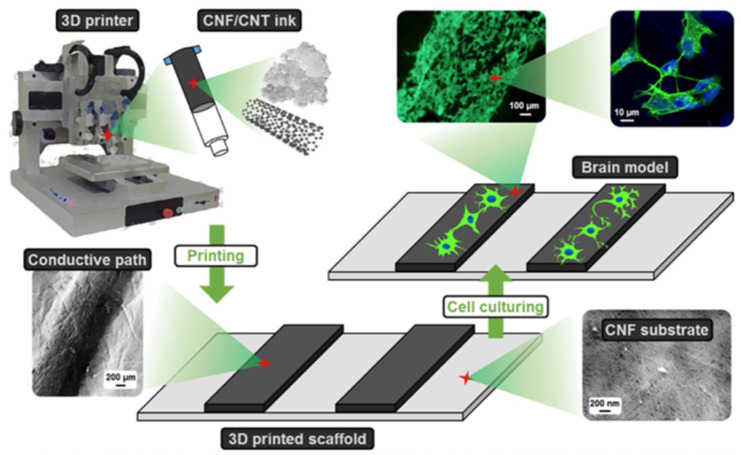
Schematic illustration of the fabrication of cellulose-derived nanofiber scaffolds for neural development. Reprinted with permission from [115]. Copyright (2018), Elsevier Ltd.

In view of the good printability and high shape-fidelity of cellulose, carboxymethylated cellulose nanofibers (CNFs) and carbon nanotubes (CNTs) could be formulated and printed into a conductive scaffold for neuron cells [115]. At very low concentrations, e.g., ~2 % wt, CNFs were able to entangle with each other and form robust hydrogel networks with crucial properties (such as shear-thinning and sufficiently high yield stress) as a printable ink. CNTs exhibit positive effects on the neural cell–matrix interaction and network development. Thus, the dispersion of CNTs (negative charge) in the negatively charged CNFs solution would yield a homogenous suspension, allowing for the fabrication of electrically conductive scaffolds for neural cells. The 2% wt of CNF-CNT (single-walled carbon nanotubes) bioinks had a high storage (elastic) modulus (G′) and exhibited elastic behavior at a low shear stress. The low viscosity profile, due to repulsion-induced low aggregation of CNF-CNT fibrils, favored the stable printing. The addition of NaOH to the composite ink was able to decrease the electrostatic repulsion and stabilize the printed structure. The scaffolds were printed onto the CNF film with a filament width of less than 1 mm. A culture of human-derived neuroblastoma cells (SH-SY5Y cell lines) on the printed scaffolds demonstrated the supportiveness for neural cells. On day 1, cells seeded on the pure CNF scaffold assembled into large clusters and showed poor proliferation and migration. The cells on the CNF-CNT scaffolds were well-spread and attached onto the scaffold grids instead of associating with each other. The electrical conductivity of the CNTs was sufficient to improve viability and proliferation and promote neural tissue development, even in the absence of external stimulation. On day 10, the CNF scaffold had negligible viability, while the CNF-CNT hybrids continued to maintain a high cell viability and proliferative rate. An evaluation of the cells on the CNF-CNT scaffolds after 10 days revealed extensive network formation. Cells also exhibited the typical neuronal-like dendritic morphology. An analysis of the 27-day culture showed larger cytoplasm and an even-greater elongation of neurites, indicating neurogenic differentiation (Figure 18).

##### GelMA–Chitosan–Dextran for Osteogenesis and Wound Healing

Dual cell-laden bioinks containing a 13% *w*/*v* GelMA shell surrounding a core of chitosan (C)–dextran (D) functionalized with peptide were bioprinted to construct pre-vascularized scaffolds for wound care [116]. The shell was laden with human bone marrow-derived mesenchymal cells (hBMSCs), and the core encapsulated human umbilical vein endothelial cells (HUVECs). It was expected that the endothelial cell core provided a natural micro-vascular framework, around which hBMSCs could grow and proliferate to mimic regenerative vascularized tissues, thereby functioning as a living dressing and providing regenerative capabilities to non-healing wounds.

The intrinsic thermo-reversibility and mechanical integrity brough forth by UV-crosslinking of GelMA (in the presence of Irgacure 2959) allowed it to serve as structural support and stabilize the construct during the printing process. Chitosan–dextran (CD) hybrid hydrogels have been reported to support cell growth and differentiation and exhibit wound healing capacity. Incorporating cell adhesion and proteolytic peptides in CD hydrogels can increase the bioactivity and promote cell growth and vascularization.

**Figure 19:**
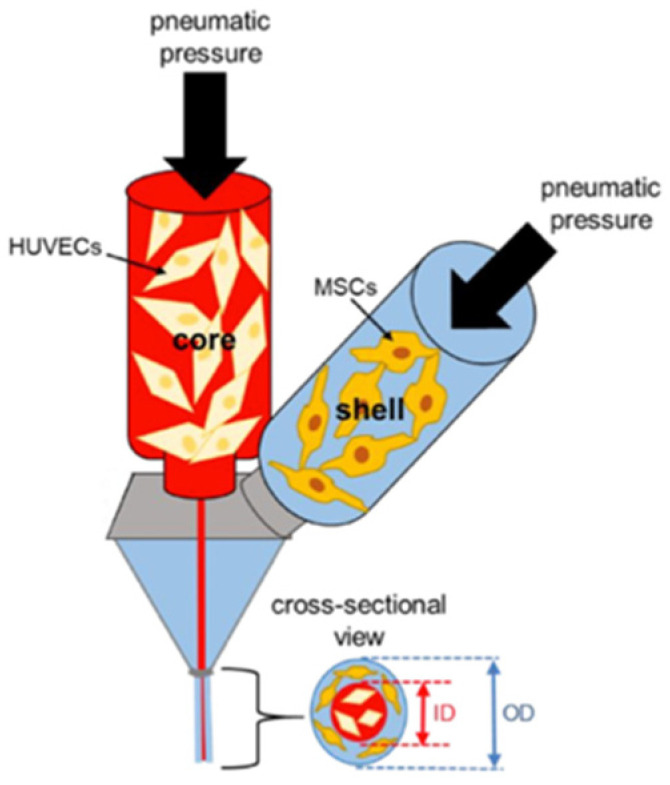
Schematic illustration of dual-head printing of GelMA shell and CD core. Reprinted with permission from [116]. Copyright (2020), American Chemical Society.

The peptide-functionalized CD hydrogel core (8% *w*/*v*), in conjunction with the GelMA shell, was printed co-axially using a core–shell extruder (Figure 19). The syringe temperature was set at 25 °C and the temperature of the print platform was set to 16 °C for the GelMA crosslinking, before irradiating each of the core/shell layers with UV light (20 s after each layer) to stabilize the printed structure.

Compressive testing showed the constructs could withstand repeated compressive cycles to 80% strain. The GelMA shell had a higher Young’s modulus (53 ± 3 kPa) over the CD core (37 ± 3 kPa). Mechanical integrity was increased in the GelMA shell. The c/s assembly was able to absorb water 3 to 4 times of their weight when immersed in PBS.

HUVECs along with hBMSCs were able to establish the vasculature and promote cell viability. Primitive cord formation was observed on day 12, and endothelial cell markers were detected on day 21. The hBMSCs also showed osteogenic differentiation.

The wound healing ability of the c/s assembly was also tested by co-culturing dermal fibroblasts (NhDFs) and keratinocytes (HaCaTs) in an in vitro wound healing model, reaching an approximately 2-fold increase of wound closure between 0 and 48 h.

Additionally, collagen-GelMA composites have played a significant role in wound healing [117]. A brain-matrix mimetic microenvironment model was constructed using HA-sodium alginate-gelatin hybrids [118] and silk composites were used to print of anatomical structures such as ear and nose [119]. These are briefed in Table 4, and a detailed description can be found in [117,118,119].

## 4. Prospective and Outlook

Biofabrication continues to make remarkable strides in the field of regenerative medicine. However, challenges such as (i) biomaterial restriction, (ii) inadequate resolution of printed structures, (iii) multi-component printing, and (iv) the lack of the physiologically functional vasculature remain to be addressed. Possible solutions to these challenges were discussed while elaborating on the foreseeable hurdles.

### 4.1. Biomaterial Limitation

During bioprinting, biomaterials not only act as support structures to the embedded cells, but also provide a 3D microenvironment for sustained cell viability and the continuous synthesis and remodeling of new ECMs for desirable tissue development [16]. However, many of the currently used biomaterials are facing many limitations in terms of their inability to meet the biological, mechanical, and technological specifications, such as biocompatibility, sufficient mechanical integrity, viscoelasticity, and photocurability [16]. In this regard, bioink formulation with multiple materials can better overcome such limitations—for example, bioinks comprising primary (matrix) and secondary phases (nanocomposites, minerals, etc., as a matrix reinforcer) to produce stronger and more durable structures while delivering biological cues to mimic the cellular microenvironment [120].

### 4.2. Lack of Printing Resolution

In view of the advantages offered by hybrid bioinks, efforts have been made to preserve the integrity, shape fidelity, and resolution of printed constructs post-printing. Interpenetrating-network (IPN) and double-network (DN) hydrogels are some examples of polymer assemblies that can give rise to self-healing hydrogels and retain the printed shape from the reversible non-covalent bonds within their networks.

**Figure 20:**
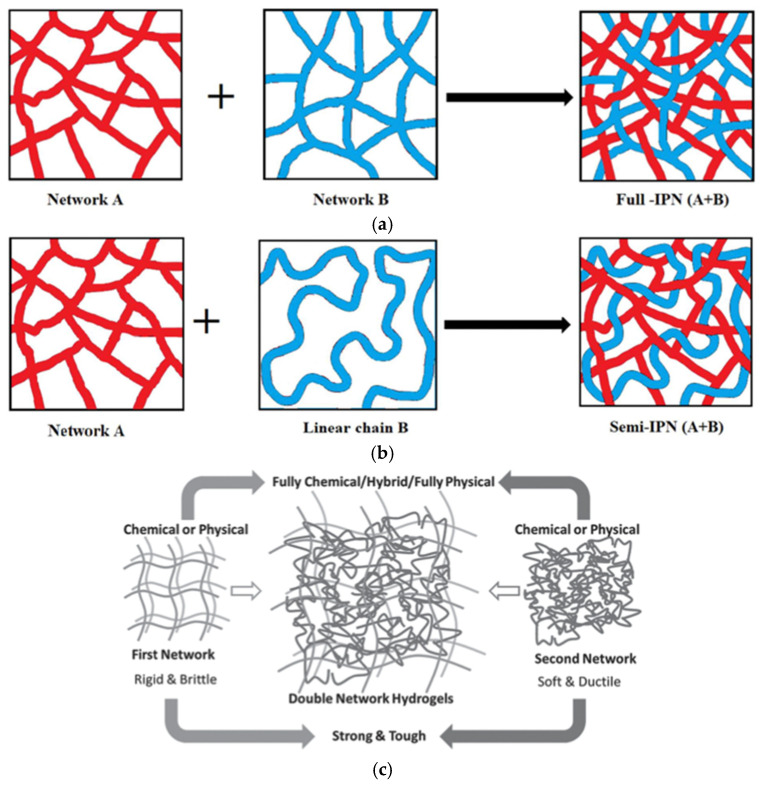
Schematic illustrations of interpenetrating-network (IPN) and double-network hydrogels. (**a**) Full-IPN; (**b**) Semi-IPN. Reprinted with permission from [124]. Copyright (2018), American Chemical Society. (**c**) DN hydrogel formation. Reprinted with permission from [125]. Copyright (2016), John Wiley and Sons.

IPN is a blend of two or more polymer networks that are interlaced, but not covalently bound [121]. Each individual polymer network retains its properties; hence, synergic improvement in toughness and strength can be seen. When one network is composed of an un-crosslinked linear polymer, it is a semi-IPN hydrogel; both individually crosslinked networks contribute to a full-IPN hydrogel, as illustrated in Figure 20a,b [121].

DN hydrogels are a special class of IPNs and are characterized by extraordinary mechanical strength, high mechanical toughness, and high water content, comparable to hard tissues such as cartilage [122,123,124]. Classical DN gels are made of polymer components with opposite physical characteristics: the first (minor) network is an abundantly crosslinked, rigid polyelectrolyte that provides the skeletal framework, and the second (major) network consists of poorly crosslinked neutral polymers that allow for network ductility (Figure 20c). The polyelectrolytic network may be replaced with a neutral network and the network formation can be commenced by introducing polyelectrolytes that act as stents to form the first crosslinked network [86]. The network structure of DN hydrogels can be tailored to create a wide variety of configurations, as detailed in [122,125]. The IPNs have been widely used in drug delivery systems. The synthesis of IPNs that act, with multi-responsive external trigger stimuli such as temperature, pH, ionic field, electric field, and light, as a way of loosening or tightening the mesh network to aid in effective drug loading and release is highly sought after. The limited swellability of the DN hydrogels indeed makes it challenging for the precisely controlled release of drugs [122,125], which can be relatively easy to achieve via the modification of IPNs or semi-IPNs.

### 4.3. Multi-Component Printing

**Figure 21:**
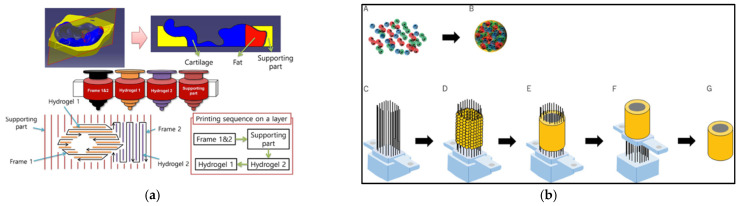
(**a**) Schematic illustration of a multi-head printing system. Reproduced from Jin Woo Jung et al. [128]. (**b**) Schematic illustration of the Kenzan printing technique. (**A**,**B**) Spheroids are prepared with only cells. (**A**) Three types of cells are prepared for spheroid formation. (**B**) Spheroid was formed and release extracellular matrix. (**C**–**G**) Spheroids are employed to fabricate scaffold-free cell construct. (**C**) Kenzan prepared for 3D bioprinting. (**D**) Spheroids are skewered onto Kenzan automatically using bio-3D printer. (**E**) Spheroids are cultured on the microneedles of Kenzan to fuse with each other. (**F**) Scaffold-free cell construct is retrieved from Kenzan. (**G**) Cell constructs are cultured on tube for further maturation. A strategy to print a heterogeneous tissue construct composed of two tissue parts and a support. Reproduced from Daiki Murata et al. [110].

Various printing techniques can also be integrated, allowing for the creation of customized printer designs to improve the printing resolution and enable multi-cell printing (Figure 21a). Modifications may include: (i) multi-armed, multi-material, multi-axis assemblies [126], (ii) inbuilt microfluidic bioink dispersion chambers to print biomaterials laden with multiple cell types simultaneously [24], (iii) tissue-on-chip platforms, with ready-made channels to aid in perfusion, and (iv) Kenzan printing (Figure 21b), in which spheroids of bioinks can be placed, one at a time, onto a microneedle array assembly. The spheroids are allowed to interact and merge with their adjacent ones to form interconnected structures [110,127].

To accommodate variations in the material properties such as viscosity for heterogenous printing, the cell–material constructs may be trapped between the biodegradable supporting frames made of thermoplastics such as polycaprolactone (PCL) or polylactic acid (PLA) (illustrated in Figure 21a). The rigid nature of these thermoplastics can help support the original print shape and avoid material fusion, even for large-volume prints [128]. The usage of materials with similar viscosity profiles is another alternative [129].

### 4.4. Vascularization

**Figure 22:**
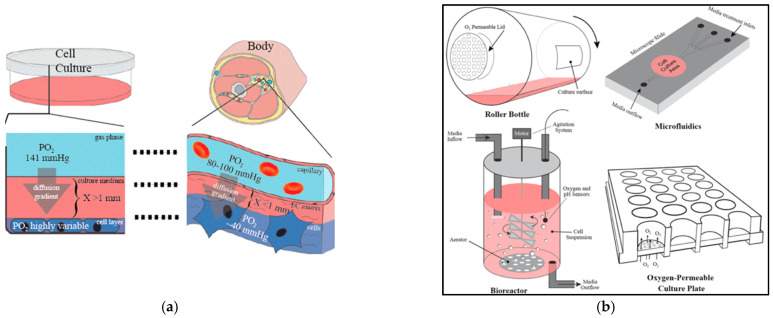
(**a**) Schematic illustration of oxygen delivery under in vitro (**left**) and in vivo (**right**) conditions. (**b**) Schematic illustration of alternates to improve O2 delivery. Spinning of the roller bottle (**top left**) on its longitudinal axis allows for oxygen diffusion between the cells and the gas that passively diffuses into the bottle through the permeable lid. In the bioreactor (**bottom left**), the well-oxygenated medium is stirred continuously by an agitator to abolish oxygen gradients. Microfluidic slides (**top right**) allow for the flow of the oxygenated medium equilibrated with precise oxygen concentrations over cells attached to the microscope slide. Oxygen-permeable plastic membranes (**bottom right**) allow the direct diffusion of oxygen from the bottom of the wells, bypassing the diffusion barrier created by the medium above and allowing for more precise control of oxygen conditions at the cellular level. Reproduced from Trenton L. Place et al. [135].

Oxygen and nutrient permeation within the hydrogel matrix occur primarily through diffusion from the growth medium (Figure 22a). Simple diffusion alone can provide oxygen for a thickness of about 100–200 μm [11,40]. However, for those thicker constructs with higher oxygen and nutrient demands, there is a need to initiate angiogenesis-like events such as native tissues, to ensure oxygen delivery to the entire structure. Diffusion limitations lead to spatial heterogeneities within the cultured constructs, characterized by significantly higher cell densities in the peripheral regions (because of a proximity to the growth medium), relative to the interior, as well as an uneven deposition of the tissue matrix due to nutrient insufficiency [11]. These limitations can be minimized through the integration of techniques such as microfluidic architecture [130], oxygen carriers [25,131], and technologies such as bioreactors [3,123], oxygen-permeable culture plates [132], and roller bottles [133] and oxygen-generating biomaterials [134] (Figure 22b), with bioprinting methodologies to enable cell culturing in well-nutritive environments. A few strategies to oxygenate tissue-engineered constructs are compared in Table 5.

## 5. Conclusions

Bioprinting has revolutionized tissue-engineering perspectives in constructing anatomically and physiologically accurate 3D models, and surpassed the traditional tissue-engineering techniques through automation, scalability, and precision. Furthermore, it also allows for the personalization of healthcare by using patient-derived cells to print de novo organs, thereby potentially reducing the organ shortage crisis while avoiding adverse immune reactions. These tissue models can also be integrated to advance drug development processes as well. While limitations still exist in the translation of biofabricated constructs beyond the laboratory workbench, unifying tissue-engineering principles with technological advancements such as efficient vascularization and innovatively formulated bioinks composed of various hydrogels and patient-derived cells can effectively and synergically converge to meet the current healthcare requirements while seeking new evolution frontiers in regenerative medicine. Hopefully, this comprehensive review of the advantages and limitations of various hydrogels can provide the foundational knowledge to facilitate the efforts in bioink formulation for tissue biofabrication.

## Figures and Tables

**Table 1:** Summary of the current bioprinting techniques.

Categories	Laser-Assisted Bioprinting (LAB)	Stereolithography (ST)	Inkjet Printing	Extrusion Printing
Energy Source	Laser beam in the UV wavelength range [14].	Light (visible and UV) [3].	Thermal, electrostatic, electromagnetic, and piezoelectric forces [14].	Pneumatic or mechanical pressure [14].
Working Mechanism	Nozzle-free printing technique that uses laser beams to direct the bioink deposition [3].	Nozzle-free procedure that uses UV or visible light [3,22].	A non-contact printing process where droplets of biomaterial are injected in the presence of an appropriate energy source [14].	Applied pressure produces continuous flow of bioink from the print nozzle [10]. Extrusion may occur through a heated nozzle (fused filament fabrication) or using external pressure (direct ink writing) [23].
Requirements	Rapid gelation, high viscosity (1–300 mPa/s) [19].	Addition of non-toxic, water- soluble photoinitiators and light absorbers to initiate photopolymerization [3].	Material must be non-fibrous in its un-crosslinked state, with a low viscosity (3.5 to 12 mPa/s) [19].	High viscosity (from 30 mPa/s to 60 × 10 ^7^ mPa/s) [19].
Common Biomaterials Used	Collagen, gelatin, fibrin, alginate [24].	Curable acrylics, epoxies [3,22].	Agarose, Matrigel [16].	Hyaluronic acid [5,16], chitosan silk [20], polyethylene glycol, poloxamers [16].
Print Resolution	10–50 μm. One cell per droplet [14].	5–300 μm [14].	50–500 μm [18].	200–1000 μm [25]
Cell Viability Rate	>95% [3].	>90% [3].	>85% [3].	As low as 40% [3].
Advantage	No clogging issues due to nozzle-free, non-contact [3,14], heterogenous cell positioning ability with high accuracy [14].	1. Clog-free, with high accuracy [3,14]. 2. Enables printing of large-scale 3D models [21].	Well suited for biomaterials with low viscosity, low cost, high printing speeds [14,19].	Good structural integrity, allows for cell printing with high densities (>10^8^ cells/mL) [25] and versatility [14].
Disadvantage	Expensive, time-consuming, low stability [3,14,24].	1. Limited due to the lack of biocompatible and biodegradable light-sensitive polymers, and the cytotoxicity of photoinitiators [3]. 2. Slow printing rate [21].	Frequent nozzle blockage [3].	Distortion in cell morphology may occur due to the high pressure needed to extrude the viscous bioink; does not allow for spatial cell positioning [3,14].

**Table 2:** Comparative summary on the hydrogel crosslinking techniques.

Categories	Physical Crosslinking	Chemical Crosslinking	Enzymatic Crosslinking
General Description	Reversible physical entanglements between polymer chains.	Robust bonding between polymers formed by the addition of external chemical agents.	Biological-derived natural catalytic factors that enable crosslinking in physiological conditions.
Crosslinkers Used	Temperature, pH, inherent molecular interactions (hydrogen, hydrophobic, and ionic bonding).	Photoinitiators (LAP, eosin Y, Irgacure), chemicals (genipin, glutaraldehyde).	Tyrosinase, transglutaminase, lysyl oxidase.
Advantages	Reversible, non-toxic.	Extremely stable, allows for control of mechanical strength.	Crosslinking is always carried under physiological conditions, and the majority of the enzymes used are common to those that catalyze in vivo reactions, are non-toxic, and can be used to crosslink opaque materials. Enzymes do not require light to be activated.
Disadvantages	Unstable and easily disrupted with changes in temperature, pH, or ionic concentration.	Crosslinkers used may induce cellular toxicity or may require additional components to be activated (e.g., Irgacure is only activated by UV light).	Crosslinking is not tunable.
Biomaterials Used	Alginate, agarose, collagen, Matrigel.	Chitosan, gelatin methacrylate, hyaluronic acid, silk.	Fibrin, gelatin, elastin, PEG.

**Table 3:** Summary of the Key Properties of Natural and Synthetic Polymers.

Polymer	Gelation Mechanism	Printing Method	Printing Concentration (*w*/*v*)	Cell Viability Rate (%)	Application	Ref.
**Natural**						
Collagen Type I	Self-assembly neutralization in acid medium + thermal gelation (37 °C), photopolymerization, chemical modification	Inkjet, extrusion	35 mg/mL	High	Cardiovascular tissue [4], Skin [49], cartilage [50], bone [51], liver [52], nerve regeneration model [53], cornea [54].	4
Fibrin	Enzymatic thrombin + CaCl_2_ + and genipin	Inkjet, extrusion	10 to 60 mg/mL	75%	Neural constructs [55], skin [56], blood vessels [57], cardiac tissues [58].	55
Hyaluronic Acid	Photopolymerization, click chemistry, chemical group functionalization (thiol, methacrylate etc.), crosslinking agents (gold, PEG)	Extrusion	2.5%	64.4 ± 12.2% (21 days)	Bone and cartilage engineering [5], tumor models [59].	5
Alginate	Divalent ions	Inkjet, extrusion, laser	1.5 to 3%	High (90 to 95%) (10 weeks)	3D-printed ear [9], vascular tissue [60], and bone printing [61].	9
Agarose	Thermal crosslinking (31 to 36 °C)	Inkjet, extrusion	0.3%	97%	Arterial bifurcation [29]	29
Chitosan	Chemical crosslinking Schiff-base reaction (genepin, glutaraldehyde) photo crosslinking	Extrusion	90:10 (ratio of chitosan to EDTA- modified chitosan)	95.6 ± 1.3% (36 h)	Cartilage engineering [62], drug delivery [63], wound repair [64].	62
Silk	Physical crosslinking (hydrophobic, hydrophilic, and hydrogen-bonding interactions), photo polymerization, enzymatic (horseradish peroxidase)	Extrusion	30% (chemically modified by methacrylate)	High (4 weeks)	Tracheal ring [65].	65
Gelatin	Thermal gelation (4 °C), chemical crosslinking by Schiff-base reaction (glutaraldehyde), photopolymerization	Extrusion	10%	78.57 ± 3.57% (Day 8)	Bioprinted ovaries [66].	66
Gelatin Methacrylate	Photo crosslinking, ionic interactions	Digital light printing, extrusion	13.3%	High (Day 10)	Nerve guidance conduit [67].	67
Matrigel	Thermal gelation (37 °C)	Inkjet, laser	2%	100% (72 h)	Co-cultures of ovarian tumor and human fibroblast cells [68].	68
**Synthetic**						
PEG	Physical, chemical, photo crosslinking	Extrusion	20%	High	Alveolar model [69].	69
Poloxamers (Pluronic (F-127))	Self-assembly (thermal gelation > 37 °C), photo crosslinking	Extrusion	20% (17% pure F-127, 3% acrylated F-127)	86.3% (Day 14)	Chondrocyte culturing [70].	70
Silicone	Chemical crosslinking	Inkjet, extrusion	Commercially available silicone (Ecoflex 50, Ecoflex30)	High (120 h)	Meniscus implants [71].	71

**Table 4:** Summary of the Composite Bioinks.

Composite	Bioink Formulation Techniques	Printing Method	Optimal Polymer Concentration	Cell Viability	Application	Ref.
Collagen–gold nanowires (GNWs)	Self-assembly after neutralization (collagen) + genipin (GNWs).	Extrusion	5% Collagen	>90% (21 days)	Muscle tissue repair	109
Agarose– Laponite	Mixing in distilled water + autoclaving (115 °C).	Extrusion	3% Agarose; 2–3% Laponite	High (7 days)	Fibroblast culturing	82
Alginate– poloxamer	Self-assembly above 37 °C (Pluronic) + divalent ions (alginate).	Extrusion	6% Alginate; 13% Poloxamer (F-127)	83 ± 6% (Day 7), full-sized tracheal ring (Day 35)	Cartilage formation	111
PVA–chitosan (CS)	Mixing in distilled water (PVA) + dissolution in acetic acid and distilled water (CS).	Extrusion	13% PVA; 1, 3 or 5% Chitosan	80–90% (7 days)	Bioprinted cornea	112
Silk–gelatin	Dissolution in 37 °C, enzymatic crosslinking (mushroom tyrosinase).	Extrusion	5% Silk; 5% Gelatin	96% (>28 days)	Skin bioprinting	113
Matrigel–agarose	Thermal self- assembly (4 °C Matrigel, 37 °C agarose).	Extrusion	50% Matrigel; 3% Agarose	77% (6 days)	Intestinal model	114
Cellulose–carbon nanotubes	Aqueous dispersion with NaOH	Extrusion	2% Cellulose; 2% CNT	High	Neural development	115
GelMA–chitosan-dextran	UV, Irgacure (GelMA) + PBS dissolution (chitosan, dextran)	Extrusion	13% GelMA; Chitosan-Dextran 8%	High	Wound healing	116
Collagen–GelMA	Enzymatic tyrosinase (collagen) + UV, Irgacure (GelMA).	Extrusion	8% Collagen; 5% GelMA	94% (14 days)	Skin wound repair	117
HA–Sodium Alginate (SA)–Gelatin (GA)	Homogeneous blending in deionized water (HA, SA, gelatin) + CaCl_2_ (SA).	Extrusion	2% HA; 1% SA; 7.5 % GA	85% (14 days)	Brain microenvironment mimetic model	118
Silk Composites	Grinding silk nanofibers dispersed in water and adding to the solution of composites such as HA, PVA and chitosan.	Extrusion	1%	92% (6 days)	Fibroblast culturing, anatomical model printing (ear, nose)	119

**Table 5:** Comparison analysis of the engineered strategies to improve oxygen delivery.

Categories	Perfusion Channels [40]	Perfluorocarbons (PFCs) [41]	Peroxides [42]
General Description	Uses microfluidics to construct perfusable networks within printed constructs.	PFCs are non-toxic, chemically inert, immiscible fluids with high oxygen and carbon dioxide transportability.	Peroxides are oxygen generators upon ready decomposition.
Mechanism of oxygenation	The perfusable channels are made of sacrificial materials to allow for mass O2 and nutrient exchange and later for guided development of blood vessels.	PFCs are hydrocarbon structures having fluorine or halogen substitutes in place of hydrogen within the polymer backbone. Being an electron-acceptor, fluorine can dissolve gases (e.g., O_2_) through diffusion.	Peroxides can interact with water to undergo hydrolytic decomposition and produce oxygen.
Requirements	Co-axial printing to allow simultaneous deposition of the structural bioink and the sacrificial template.	Being extremely hydrophobic with certain lipophilic characteristics, PFCs require surfactants such as lecithin to form suitable emulsions that can be incorporated within the bioink.	Peroxide decomposition into water and oxygen is related to the formation of hydrogen peroxide that is detrimental to cells. The incorporation of catalase enzyme within the printed bioink, along with the oxygen-generating peroxides.
Need	Inducement of angiogenesis through growth factors requires long intervals to establish functional vasculature during which mass transport may be compromised due to diffusion limitation in the thicker structures [12]. Pre-vascularization offers immediate oxygen and nutrient perfusion, by-passing the time lag associated with vasculature formation.	In view of the high molecular ratios of dissolved O_2_ in PFC (5O_2_:1PFC), 1000-times higher than water, the incorporation of PFCs within bioinks can help attract and direct oxygen from the growth medium to the cells encapsulated within the printed construct to better oxygenate the cells.	Incorporation of peroxides within bioinks can assist in timely decomposition of hydrogen peroxide decomposition and maintain cell viability within the printed construct (as seen in the pictorial representation below) [134].
Examples	Sacrificial channels can be made from temperature-sensitive biomaterials such as gelatin, GelMA, or Pluronic.	Perfluoro-octyl bromide (PFOB) and perfluoro-decalin (PFD) can be used.	Calcium, magnesium, or sodium peroxides can be used.
Efficiency	Co-fabrication of perfusable vascular channels has seen improved cell survival (maintained 80% viability over a 14-day period) and function within the printed structures [40].	Molecular ratios of dissolved O_2_ are 1O_2_:200water in water, but 5O_2_:1PFD in PFD, resulting in a 1000-times increased molecular solubility of O_2_ for PFD compared to water (as seen in pictorial representation below).	Cell viability analysis on day 7 after incorporating 1% calcium peroxide is 80% [134].
Pictorial Representation	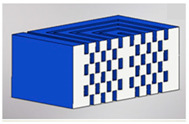	* 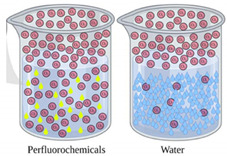 *	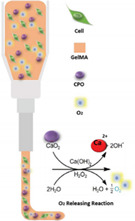

## Data Availability

Not applicable.
